# Marine Biodiversity in the Caribbean: Regional Estimates and Distribution Patterns

**DOI:** 10.1371/journal.pone.0011916

**Published:** 2010-08-02

**Authors:** Patricia Miloslavich, Juan Manuel Díaz, Eduardo Klein, Juan José Alvarado, Cristina Díaz, Judith Gobin, Elva Escobar-Briones, Juan José Cruz-Motta, Ernesto Weil, Jorge Cortés, Ana Carolina Bastidas, Ross Robertson, Fernando Zapata, Alberto Martín, Julio Castillo, Aniuska Kazandjian, Manuel Ortiz

**Affiliations:** 1 Departamento de Estudios Ambientales, Universidad Simón Bolívar, Caracas, Venezuela; 2 Centro de Biodiversidad Marina, Universidad Simón Bolívar, Caracas, Venezuela; 3 Universidad Nacional de Colombia, Bogotá, Colombia; 4 Universidad de Costa Rica, El Centro de Investigación en Ciencias del Mar y Limnología (CIMAR), San José, Costa Rica; 5 Museo del Mar, Margarita, Venezuela; 6 Department of Life Sciences, University of West Indies, St. Augustine, Trinidad and Tobago; 7 Universidad Nacional Autónoma de México, Instituto de Ciencias del Mar y Limnología, Mexico City, Mexico; 8 Department of Marine Sciences, University of Puerto Rico, Lajas, Puerto Rico; 9 Departamento de Biología de Organismos, Universidad Simón Bolívar, Caracas, Venezuela; 10 Smithsonian Tropical Research Institute, Balboa, Ancón, Panamá; 11 Department of Biology, Universidad del Valle, Cali, Colombia; 12 Centro de Investigaciones Marinas, Universidad de La Habana, La Habana, Cuba; NIWA, New Zealand

## Abstract

This paper provides an analysis of the distribution patterns of marine biodiversity and summarizes the major activities of the Census of Marine Life program in the Caribbean region. The coastal Caribbean region is a large marine ecosystem (LME) characterized by coral reefs, mangroves, and seagrasses, but including other environments, such as sandy beaches and rocky shores. These tropical ecosystems incorporate a high diversity of associated flora and fauna, and the nations that border the Caribbean collectively encompass a major global marine biodiversity hot spot. We analyze the state of knowledge of marine biodiversity based on the geographic distribution of georeferenced species records and regional taxonomic lists. A total of 12,046 marine species are reported in this paper for the Caribbean region. These include representatives from 31 animal phyla, two plant phyla, one group of Chromista, and three groups of Protoctista. Sampling effort has been greatest in shallow, nearshore waters, where there is relatively good coverage of species records; offshore and deep environments have been less studied. Additionally, we found that the currently accepted classification of marine ecoregions of the Caribbean did not apply for the benthic distributions of five relatively well known taxonomic groups. Coastal species richness tends to concentrate along the Antillean arc (Cuba to the southernmost Antilles) and the northern coast of South America (Venezuela – Colombia), while no pattern can be observed in the deep sea with the available data. Several factors make it impossible to determine the extent to which these distribution patterns accurately reflect the true situation for marine biodiversity in general: (1) highly localized concentrations of collecting effort and a lack of collecting in many areas and ecosystems, (2) high variability among collecting methods, (3) limited taxonomic expertise for many groups, and (4) differing levels of activity in the study of different taxa.

## Introduction

### Physical and geological description of the Caribbean

**Figure 1:**
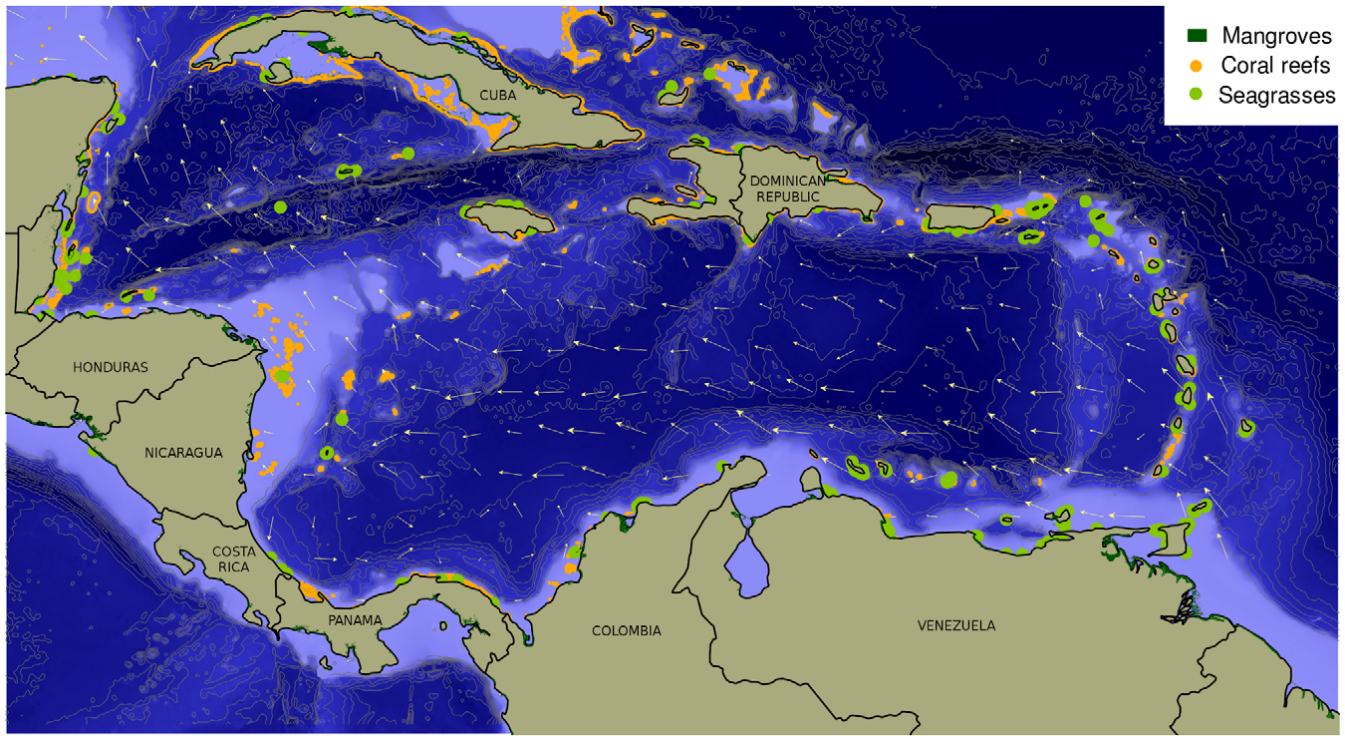
Bathymetry, main currents, and ecosystems of the Caribbean Sea. Arrows representing average surface ocean currents were derived from the Hybrid Coordinate Ocean Model, or HYCOM (http://hycom.org). Coral reef data were obtained from the World Resources Institute (http://www.wri.org/publication/reefs-risk-caribbean). Data on seagrasses were extracted from version 2.0 of the global polygon and point dataset compiled by UNEP World Conservation Monitoring Centre (UNEP-WCMC), 2005. Mangrove data were extracted from version 3.0 of the global polygon dataset compiled by UNEP-WCMC in collaboration with the International Society for Mangrove Ecosystems (ISME), 1997.

The Caribbean Sea is a semienclosed basin of the western Atlantic Ocean, bounded by the coasts of Central and South America on two sides and by the Antilles island chain on the other two (Figure 1). It has an area of about 2,754,000 km^2^, a volume of nearly 6.5×10^6^ km^3^, and over 13,500 km of coastline, and is home to 26 countries as well as 19 dependent territories of France, the Netherlands, the United Kingdom, and the United States. Toward the east and northeast, the closely spaced chain of islands, banks, and sills of the Antilles Islands arc separates the Caribbean from the Atlantic Ocean and acts as a sieve for the inflow of Atlantic water [1], whereas toward the northwest the Caribbean is linked to the Gulf of Mexico by the Yucatan Channel. The Caribbean seafloor is divided into five basins (Grenada, Venezuela, Colombia, and Yucatan Basins and the Cayman Trough) separated from each other by underwater ridges and sills. Half of the waters in the Caribbean are deeper than 3,600 m, and 75% are deeper than 1,800 m [2]. The average seafloor depth is about 2,400 m, while the Cayman Trough, between Cuba and Jamaica, reaches more than 7,500 m [3]. Volcanic activity and earthquakes are common in the Caribbean, as are destructive hurricanes, most of which originate in the central Atlantic.

The Caribbean has an overall counterclockwise circulation (Figure 1). The Caribbean Current enters the southeast corner of the basin through several passages of variable sill depth between the Lesser Antilles and, to a lesser extent, the Windward Passage, and slightly increases its velocity as it flows west-northwesterly into the Gulf of Mexico through the Yucatan Channel, where it forms the Gulf Stream (see [4]). Caribbean waters are mostly clear and warm (22–29°C), and the tidal range is very low (<0.4 m) [5]. The water column is highly stratified in the upper 1,200 m because of the sill depths of the Antilles Islands arc, which prevents the flow of deep water into the Caribbean Basin [6]. The Caribbean geology was recently reviewed by Jackson [7]. The deep Caribbean Sea evolved by seafloor spreading since the Jurassic, but the key aspects of the tectonic history have been subject to controversy [8], [9]. Two models explain the late Mesozoic formation and the evolution of the Caribbean Plate. The first suggests that the Caribbean crust was formed between the South American and North American plates (model reviewed in Meschede and Frisch [10]). The second suggests a late Mesozoic origin of the Caribbean crust in the Pacific region as a result of a flood basalt event at the Galapagos hot spot and a later drift to the east during the Cenozoic times [11]–[13]. Meschede and Frisch [14], concluded from geological, geochronological, and paleomagnetic evidence that the Caribbean crust was originally formed in an inter-American position (adjacent to the northwestern margin of South America) during the middle to upper Cretaceous, not in the Galapagos hot spot, and that the source for the Caribbean flood basalt must be a plume between the two Americas that was active during the middle and upper Cretaceous. The Eocene basalt and the pelagic cover formed a relatively deep floor in which arc-derived turbidites and pelagic sediments have accumulated over 25–30 millions of years.

The ratio of continental margin to total open ocean area in the Caribbean basin is larger than in the major ocean basins, meaning that the margins have greater potential importance to physical, geological, and biological processes. Major river systems and associated features characterize the seafloor on the continental shelf and influence the offshore habitats with sediment input. The coastal ecosystems in the Intra Americas Sea (IAS) are highly productive in contrast to the oligotrophic offshore waters, and are mainly characterized by particulate organic carbon (POC) flux. In offshore waters the pelagic deposition and turbidity currents have been correlated with the benthic macrofaunal standing stock [15]. With the exception of restricted turbid coastal areas near rivers, the most salient feature of the IAS is its warm, transparent water, compared to other large ocean systems. This water clarity is a function of the oligotrophic conditions and strong influence of oceanic water masses in the region. The Orinoco plume spreads widely over the Caribbean affecting significantly the optical properties of the water in the eastern Caribbean Sea by introducing large amounts of colored dissolved organic matter and nutrients and thus increasing primary productivity [16]. Elevated pigment concentrations are visible within the southern Caribbean where the shoaling of the deep chlorophyll maximum and dispersal of the water mass occur [17]. The sediment and organic matter particles transported from the Orinoco and Amazon rivers by the northward moving Guiana current enter and disperse in the Caribbean Sea and the near Atlantic [18] and are deposited on the western flank of the Aves Ridge. Higher zooplankton production in the southeastern Caribbean may also enhance transport of organically rich suspended matter into fecal pellets that have accelerated sinking rates (Richardson et al 1995). This labile organic matter raining from the overlying water is tightly coupled with the benthic assemblages in the Venezuela Basin [19]. Based on penetration profiles done with an echosounder, a continuous sedimentation from the water column to the seafloor characterizes the Puerto Rico Trench. The top 10 cm of this sediment is brown to brownish gray and shows evidence of both coastal and pelagic input [20].

The most characteristic ecosystems in the Caribbean are coral reefs covering about 26,000 km^2^ [21], seagrass beds with an area of about 66,000 km^2^ [22], and mangroves at nearly 11,560 km^2^ [23]. Although the Caribbean has been considered as oligotrophic, it can be better defined as mesotrophic, depending on the time of the year [24]. The intrusion of the Orinoco River during autumn generates large concentrations of chlorophyll a in the eastern Caribbean, which can be carried up to the island of Puerto Rico [24]. Moreover, strong trade winds during winter and spring are responsible for coastal upwelling along much of the coastline of northeast Colombia and Venezuela, bringing nutrients to the surface and increasing the concentration of phytoplankton biomass in that area [1], [25]. The deep-sea habitats in the Caribbean Sea and surrounding areas include typical abyssal soft-sediment extensions, numerous small canyons [26], and wide (10 km) and low-relief (<5 m) channels over the Orinoco deep-sea fan, an accretionary prism near Barbados [27], seamounts (e.g., Niobe, Chía, Ubaté, Calima), ridges (e.g., Tayrona, Aves), hills (e.g., Aracataca, Tumaco, Pijao), tablemounts (Explorer) where encrusting coralline algae have been recorded at 268 m depth in the Bahamas [28], knolls (e.g., Naquí, Nectier), and escarpments (Hess). According to the latest marine biogeographic classification system into marine ecoregions (Marine Ecoregions of the World or MEOW) [29], the Greater Caribbean is part of the Tropical Northwestern Atlantic Province which comprises nine ecoregions: Western Caribbean, Southwestern Caribbean, Eastern Caribbean, South Caribbean, Greater Antilles, Bermuda, Bahamian, Southern Gulf of Mexico, and Floridian. In this paper, we will focus on the first five ecoregions, which include the entire Caribbean basin and the north of the islands of the Greater Antilles. The other four ecoregions are developed in another article in this collection [30].

### History of exploration of marine biodiversity in the Caribbean

Species inventories are the most elementary data in ecology, biogeography, and conservation biology. Species records are mostly used to determine the number of species occurring in a given area, but they can also be employed to determine distribution patterns, for the identification of biodiversity “hot spots” or for designing conservation strategies [31], [32].

Species extinction has reached unprecedented rates on both land and ocean [33], [34], and these rates are much higher than those of new species discovery [35], [36]. This suggests that there is a crisis in global information or, in other words, a critical weakness in the world's “knowledge economy” [37]. The increasing human impacts on marine biodiversity and the need to optimize and set priorities among limited resources for implementing conservation measures have impelled the description of diversity patterns and, consequently, have encouraged the use of taxonomic inventories [38], [39]. However, the use and comparability of species inventories are limited by the extent of their completeness and the heterogeneity of sampling effort between sites or areas [40].

The Caribbean contains the greatest concentration of marine species in the Atlantic Ocean and is a global-scale hot spot of marine biodiversity [32]. Because the Caribbean is regarded as a distinct biogeographic province of the Tropical Western Atlantic Region, several authors (e.g., [29], [41]–[46]) have proposed more detailed biogeographic regionalizations within the Caribbean, using a variety of criteria for defining divisions.

The growth of human population, particularly in coastal zones, and the environmental pressures imposed by economic growth and climate change pose great challenges to the future conservation of marine ecosystems and species diversity. In particular, the Caribbean Sea has large population densities, a long history of human use of marine resources, and remarkable land-based sources of pollution associated with oil production, port and tourism development, deforestation, and agriculture [45], [47]. The areal coverage of mangroves in the Caribbean has decreased by about 1% per year since 1980 [47]. Live coral cover has already declined by as much as 80% in many areas of Caribbean reefs over the last two decades because of various human activities and global warming [48], [49], and 35% of the region's fish stocks are overexploited [50], [51]. Despite a long history of scientific research in the region, our present knowledge about Caribbean marine biodiversity and species distribution does not satisfy the needs for objectively defining geographic conservation priorities and designing management plans at a regional scale. This is one of the reasons why conservation planners often make use of surrogates of species diversity (e.g., presence of habitats, bottom topography, wave exposure) to offset uncertainty and lack of detailed information (see [52], [53]), as has been the case in various recent attempts to determine the relative importance of sites for conservation in the Caribbean (e.g., [54]–[56]).

Although the first scientific expedition to the New World did not occur until 1799, species discoveries and descriptions of marine organisms from the Caribbean started several years earlier from preserved fishes, coral skeletons, and mollusk shells collected during exploration voyages. These samples were transported to Europe, where they were described and deposited in museum collections. With a few exceptions, such as Charles B. Adams (1814–53) and William Stimpson (1832-72), who spent several years collecting and studying marine mollusks in the Caribbean, most of the taxonomists who described marine species from the Caribbean until the end of the nineteenth century were land based, working in museums or universities. Such taxonomists seldom collected specimens themselves in the field and had a limited knowledge of the distribution and ecology of the samples they received. With only a single specimen of each species, a shell, or a fragment of a colony, these naturalists worked with a magnifier, a lamp, paper, ink, and a pen on a bench, with approximate information about the locality where the specimen was found. Local and geographic morphological variability could not be assessed. Nevertheless, they did a remarkable job with their descriptions and drawings. By the beginning of the twentieth century, about half of the marine species known today from the Caribbean had already been described.

A remarkable impulse to the knowledge of Caribbean species diversity, particularly from deep waters, stemmed from several scientific cruises conducted in the late nineteenth century. In particular, the cruises of the U.S. Coast Survey vessel *Blake* (1877-80), under the scientific supervision of Alexander Agassiz, became a pivotal event in the exploration of the Gulf of Mexico and the Caribbean; hundreds of species of hydroids, corals, antipatharians, crustaceans, echinoderms, annelids, mollusks, fishes, and other organisms from depths of up to 3,000 m were described based on the collections obtained from the *Blake* expeditions. A second impetus in Caribbean species inventories occurred in the 1970s with the advent of scuba diving and more extensive collections that greatly helped to refine the taxonomical classifications and increase the knowledge of the taxonomy, ecology, and variability of many groups.

### Research capacity in the Caribbean region

Most Caribbean countries (with the exception of Haiti, Honduras, Guatemala, Nicaragua, and some of the small insular states) have well-known marine research stations and laboratories, which are usually tied to academic institutions with long-standing traditions in the study of marine organisms. The majority of them, despite different cultures, financial resources, and capabilities, have developed a common interest in cooperation and networking since the 1980s, through national and local government departments and nongovernmental organizations (NGOs); universities and other tertiary learning institutions; regional intergovernmental organizations (IGOs); UN organizations; and international NGOs.

The first regional marine science organization in the Caribbean, the Association of Marine Laboratories of the Caribbean (AMLC), was established in 1957 by nine research institutions. It evolved into a confederation of more than 30 marine research, education, and resource management institutions and more than 300 individual members. Its main objective is to encourage the production and exchange of research and resource management information, to advance the cause of marine and environmental education in the region, and to facilitate cooperation and mutual assistance among its membership (www.amlc-carib.org/). One of the most successful research programs developed on the strength of the AMLC was the Caribbean Coastal Marine Productivity Program (CARICOMP), which was a regional scientific program supported by UNESCO and the U.S. National Science Foundation. The aims of this program included the monitoring of long-term changes in the three main coastal ecosystems in the Caribbean region—mangroves, seagrass beds, and coral reefs, while it left the offshore and deep-sea habitats remaining poorly documented. Monitoring activities and data collection began in 1992 at 29 sites in 22 countries and territories, using standard research methods, building regional capacity and shared expertise (http://www.unesco.org/csi/act/caricomp/summary14.htm). Data was archived at the CARICOMP Data Centre at the University of the West Indies in Jamaica. While this program has formally ended, at present, CARICOMP monitoring activities still take place in Colombia, Panama, Costa Rica, and Venezuela, among other sites. Currently, discussions are being held within the AMLC about the need to continue a regional monitoring program.

Another remarkable regional scientific initiative in the region includes the Cooperative Investigations of the Caribbean and Adjacent Regions (CICAR) dating back to the 1970s. Its aim was to develop capabilities among the participating countries to carry out marine scientific research and the understanding of oceanographic processes in the Caribbean region [2]. As a successor organization to CICAR, in 1982 IOCARIBE (the Sub-Commission for the Caribbean and Adjacent Regions, of the Intergovernmental Oceanographic Commission (IOC) of UNESCO) was created, with 19 member states.

A Protocol of the Convention for the Protection and Development of the Marine Environment of the Wider Caribbean Region came in effect into 1986 to protect the endangered marine life of the Caribbean by prohibiting human activities that would result in the continued destruction of such marine life in various areas. The protocol has been ratified by 15 countries and diverse NGOs, such as The Nature Conservancy, the World Wildlife Fund, and the Caribbean Conservation Corporation, that have been involved in the preservation of Caribbean marine life. The Census of Marine Life (Census) program became involved in the region in 2004 with the Caribbean Marine Biodiversity Workshop. In this workshop, 10 of the largest Caribbean countries reviewed the status of knowledge of marine biodiversity within their boundaries (Venezuela, Colombia, Panama, Costa Rica, Mexico, Bermuda, Cuba, Jamaica, Puerto Rico, and the Dominican Republic), resulting in the production of a regional report (see [39]). The workshop also led to a productive interaction between researchers, conservation agencies, and oil companies, which established links for international collaboration and future partnerships within the Census umbrella. The main projects that the Census advanced in the Caribbean region were History of Marine Animal Populations (HMAP), Natural Geography in Shore Areas (NaGISA), Continental Margin Ecosystems on a Worldwide Scale (COMARGE), and Biogeography of Deep-Water Chemosynthetic Ecosystems (ChEss). These projects carried out field work in several sites in the region and the data can be found in the Ocean Biogeographic Information System (OBIS). The most recent of these expeditions (April 2010) was the British cruise on the Royal Research Ship *James Cook* to the Cayman Trough, the world's deepest undersea volcanic rift, which runs across the Caribbean seafloor. Besides these projects, the region also participated in Antarctic research with the Census of Antarctic Marine Life (CAML) project. The Caribbean region also contributed substantial amounts of data to the Ocean Biogeographic Information System (OBIS) database and proposed a sister project to the established Census of Coral Reef Ecosystems (CReefs), aimed to update and clarify the taxonomy and distribution of the major benthic coral reefs groups. In addition, it established a network of researchers associated with the International Census of Marine Microbes (ICoMM) project. Besides contributing significantly to the knowledge of marine biodiversity in the Caribbean region, the Census established regional networks for scientific cooperation.

In 2005 the United Nations General Assembly endorsed the need for a regular process for global reporting and assessment of the state of the marine environment. The “Assessment of Assessments,” begun during the start-up phase of the process, has as its main objective an overview of the geographic and thematic coverage of existing assessments on oceans and coastal areas at regional and global levels. The assessment established the relative importance of issues being assessed in the region and analyzed the capability of the region to undertake future assessments of issues that have clear links to neighboring regions. These future assessments include biodiversity, ecosystems (corals, mangroves, seamounts), mammals, genetic resources, and invasive alien species. The spatial framework developed for the Assessment of Assessments is based on both biogeographic factors and administrative structures conducive to an ecosystem approach. The Caribbean Sea was recognized as such an entity for that assessment.

Here we analyze the state of knowledge of Caribbean marine biodiversity using georeferenced species-record data and species lists for localities within that region. Our first goal is to analyze spatial heterogeneity of the data to determine gaps in knowledge and the effect of biases in the distribution of geographical data within the established ecoregional biogeographical divisions in the Caribbean. Our second goal is to assess patterns in the distribution of members of these five groups of marine organisms and test if species distribution actually fits to the biogeographic model of the five ecoregions proposed. The paper also discusses the role of the Census of Marine Life program in advancing knowledge about marine biodiversity in the Caribbean as well as the major threats to marine biodiversity in the region. Our hope with this paper is to increase awareness of the value of taxonomic inventories and of how much and where scientific sampling is needed to understand better the large-scale geography of Caribbean marine biodiversity.

## Methods

To compile available data on marine species diversity in the Caribbean, we used two approaches. The first approach was to summarize the number of species for all taxonomic groups using georeferenced species records from open-access databases (especially OBIS) and from local, country, territory, and regional checklists. The second approach was to produce revised species lists for the relatively well known taxonomic groups (sponges, stony corals, polychaetes, mollusks, amphipods, and echinoderms) by country or subregions (where there is information available). Only taxonomically valid species were included, based on the expertise of taxonomist authors of this paper. Introduced and invasive species were also incorporated. We also reviewed records of the distribution of shallow-water shore fishes within the Caribbean, as well as deep-sea records (below 200 m depth) for all taxonomic groups. The main data sources used for constructing these matrices are presented in Table 1, which lists the essential literature for marine biodiversity studies in the Caribbean.

**Table 1:** Sources of data used to estimate total number of marine species for different taxa and for the deep sea.

Taxa/Environment	Literature, museum and database sources
**Algae**	[159]–[162]Museums: HNV, MMMDatabases: www.obis.org
**Porifera**	[72], [89]–[92], [114], [163]–[187]Databases: www.marinespecies.org/porifera / www.spongeguide.org / www.obis.org
**Scleractinia**	[95], [186], [188], [189]–[202]Databases: www.reefbase.org
**Polychaeta**	[203]–[207]
**Mollusca**	[137], [208]–[220]Museums: MHNMC, NMNHDatabases: www.malacolog.org / www.sealifebase.org / www.marinespecies.org www.cephbase.utmb.edu / www.redciencia.cu/cdbio/Contenido www.jaxshells.org/cayman.htm / www.jaxshells.org/abc.htm
**Amphipoda**	[221]–[239]Museums: GCCAS, USB-ANF, UMML, MNCN, MBUCV, ZMA, BMNHDatabases: www.obis.org
**Echinodermata**	[82], [105], [107], [109], [240]–[279]Museums: GCCASDatabases: www.itis.gov
**Pisces**	[115], [280]–[289]Museums: NMNHDatabases: www.obis.org / Personal database of coauthors DRR and FAZ
Deep sea (*)	[251], [261], [263], [274], [279], [290]-[297]

GCCAS: Geology Collection of the California Academy of Sciences, San Francisco, USA.

USB-ANF: Collection of Peracaridean Crustaceans – Amphipods from Museo de Ciencias Naturales – Universidad Simón Bolívar, Caracas, Venezuela.

UMML: Marine Invertebrate Museum, Rosenstiel School of Marine and Atmospheric Science, University of Miami, USA.

MNCN: Museo Nacional de Ciencias Naturales, Madrid, Spain.

MBUCV: Museo de Biología de la Universidad Central de Venezuela, Caracas, Venezuela.

ZMA: Zoological Museum of Amsterdam, Amsterdam, The Netherlands.

BMNH: British Museum of Natural History, London, UK.

HNV: Herbario Nacional de Venezuela, Caracas, Venezuela.

MMM: Museo del Mar, Isla de Margarita, Venezuela.

MHNMC: Museo de Historia Natural Marina de Colombia, INVEMAR, Santa Marta, Colombia.

NMNH: National Museum of Natural History, Washington D.C., USA.

(*)The deep-sea review encompasses 1,530 species grouped in 12 phyla [Porifera, Cnidaria, Chaetognata, Mollusca, Sipunculida (still considered separate from Annelida), Annelida (subdivided into Polychaeta and Echiura), Bryozoa/Ectoprocta, Brachiopoda, Pycnogonida, Crustacea, Echinodermata, and Cephalochordata (demersal fish only)].

In order to test the hypothesis of different species composition assemblages for each of the five marine ecoregions of the Caribbean area (as proposed by Spalding et al. [29]), we used a permutational multivariate ANOVA [57] as implemented in R package “vegan” [58]. Using the country presence-absence matrix for each taxonomic group, we estimated a dissimilarity matrix based on Sorensen's index, and then each country was recoded as a member of its particular ecoregion. If the ecoregional pattern for a particular taxa represents different species assemblages, a statistically different community ordination should result in the analysis. A graphical representation of the ordination was made using a non-metric multidimensional scaling, so countries within the same marine ecoregion would be expected to group closely in the MDS. Countries or territories considered within each of the marine ecoregion were: (1) *Western Caribbean*: Mexico, Belize, Honduras, Guatemala, (2) *Southwestern Caribbean*: Nicaragua, Costa Rica, Panama, Colombia, San Andres Island, (3) *Southern Caribbean*: Venezuela, the Netherland Antilles (Aruba, Bonaire and Curacao), Trinidad and Tobago, (4) *Greater Antilles*: Cuba, Jamaica, Puerto Rico, Dominican Republic, Haiti (these last two together are also known as Hispaniola Island), Cayman Islands, (5) *Eastern Caribbean*: Barbados, Virgin Islands, and combined information from several of the islands comprising the Lesser Antilles. To analyze the relative contribution of each ecoregion to the Caribbean regional diversity (gamma diversity), we used the contribution partition analysis proposed by Lu et al. [59]. Each ecoregion's species list (richness) represents the ecoregional diversity. According to the partition of species diversity, where the regional (gamma) diversity is the sum of the local (alpha) and interlocal (beta) diversity, an index of relative contribution of each term could be estimated. For each ecoregion, the greater the number of species that are listed, the higher its alpha diversity. However, depending on the number of endemic or exclusive species in an ecoregion, the relevance of this ecoregion to the relative contribution to the gamma diversity could change. The same analysis was done for individual countries to determine which of the countries within each ecoregion contributed more to the gamma diversity. For example, a country or subregion with few species, many of which are endemic (or exclusive), contributes more to the regional diversity than a country with many but wide-ranging species.

Additionally, to assess whether or not the rate of discovery of a particular type of fauna shows a tendency to decline, thus indicating that we are approaching its full description, we established the number of new species described per year and plotted accumulation curves for fishes, mollusks, and echinoderms. Since the quality of taxonomic inventories depends strongly on the availability of identification guides and taxonomic experts, our review also included an account of these resources for each taxonomic group (Table 1).

## Results

### Taxonomic inventories

At least 12,046 species have been reported to occur in the Caribbean Sea (Table 2, Table S1). These include representatives from 31 animal phyla, two plant phyla (green and red algae and Angiospermae: mangroves and seagrasses), one group of Chromista (brown algae), and three groups of Protoctista (Foraminifera, Dinoflagellata and Amoebozoa). The quality of information available differs considerably among these taxa, and only poor information is available on bacteria, Cyanophyceae, and diatoms (Chrysophyta). For the Dinoflagellate (Pyrrhophyta), 85 invertebrate species within the Anthozoa, Hydrozoa, Scyphozoa, Actinaria, Gorgonacea, Zoanthidae, Corallimorpharia, and Gastropoda have been reported to have one or more clades of the symbiont dinoflagellate *Symbiodinium*, with a total of 31 different clades (Table 3, Table S2). For many taxonomic groups, the number of known species is constantly increasing as new species are described or are recorded for the first time in the region. Knowing the taxonomic background (availability and expertise) of the region, we had not expected to be able to produce species lists of the same quality for the different taxonomic groups. However, for most of the groups, our review can be considered satisfactory. We consider only 16 of the 78 (about 20%) species counts at the phylum to order level (Table 2 and Table S1) deficient in quality or incomplete: Fungi, Placozoa, Entoprocta, Brachiopoda, Phoronida, Nemertea, Gnathostomulida, Pogonophora, Rotifera, Priapulida, Kinorhyncha, Tardigrada, Nematoda, Branchiopoda (Cladocera), Ostracoda, and Urochordata. Species in these taxa represent probably less than 5% of the species reported in Table 2. The counts for the remaining groups should be considered satisfactory, with a presumable error margin of less than 5%. However, about half of these counts would greatly benefit from further taxonomic review. No species were reported from four phyla (Nematomorpha, Loricifera, Micrognathozoa, and Cycliophora), which is probably because of a lack of taxonomic attention rather than the absence of these groups from Caribbean waters. By far, the most speciose taxa are Mollusca (3,032 species), Crustacea (2,916 species), and Pisces (1,336 species), which together account for about 60% of the total biota. Mollusks are also the most diverse group for all countries and ecoregions (Table 4).

**Table 2:** Diversity, state of knowledge, and expertise of the main taxonomic groups within the Caribbean region.

Taxonomic group	No. species^1^	State of knowledge^2^	No. introduced species	No. experts	No. identification guides^3^
**Domain Archaea**	ND	ND	ND	ND	
**Domain Bacteria (including Cyanobacteria)**	5	1	ND	ND	
**Domain Eukarya**					
**Kingdom Chromista**					
Phaeophyta	71	4	ND	10	1
**Kingdom Plantae**					
Chlorophyta	170	4	2	10	1
Rhodophyta	320	3	3	10	1
Angiospermae	14	5	1	13	2
**Kindom Protoctista (Protozoa)**					
Dinomastigota (Dinoflagellata)	>31 (*1)	2	ND		
Foraminifera	704	2	ND		1
**Kingdom Animalia**					
Porifera	519	4	1	7	4
Cnidaria	994	1–3	5	20	8
Platyhelminthes	129	3	ND	2	1
Mollusca	3032	1–4	6	20	8
Annelida	658	3	2	31	1
Crustacea	2916	2–4	7	57	10
Bryozoa	131	2	2	2	0
Echinodermata	438	3–4	ND	5	2
Urochordata (Tunicata)	62	3	1	ND	1
Other invertebrates	402				
Vertebrata (Pisces) (*2)	1336	3–5	15	∼55	16
Other vertebrates	59	4–5	0	>150	10
**SUBTOTAL**	**11,991**				
**TOTAL REGIONAL DIVERSITY** 4	**12,046**		**45**	**388**	**67**

**Notes:**

1 Sources of the reports: databases, scientific literature, books, field guides, technical reports.

2 State of knowledge: 5 =  very well known (>80% described, identification guides <20 years old, and current taxonomic expertise); 4 =  well known (>70% described, identification guides <50 years old, some taxonomic expertise); 3 =  poorly known (<50% species described, identification guides old or incomplete, no present expertise within region); 2 =  very poorly known (only few species recorded, no identification guides, no expertise); 1 =  unknown (no species recorded, no identification guides, no expertise). ^3^Identification guides cited in Table 1 and in References.

4 Total regional diversity including all taxonomic groups as reported in Appendix 1.

(*1) At least 31 clades of the genus *Symbiodinium* are found in 85 species of invertebrates.

(*2) Shore fish species that occur in the upper 100 m of the water column.

ND  =  No data.

**Table 3:** Summary of *Symbiodinium* clades (Dinoflagellata) found in invertebrates sampled in the Caribbean.

Taxonomic group	Clade designation of symbiont	Reference
Anthozoa Scleractinia (47)	A, A3, A4a, B, B1, B5a, B6, B7, B9, C, C1, C1a, C2, C3a, C3c, C3e, C4, C9, C11, C12, D, D1a,	[298]–[312]
Anthozoa Actinaria (5)	A3, A4a, B1, C1	[301], [305]
Anthozoa Zoanthidae (3)	A3, A4, B1, C1, C3, D1	[301], [305], [313]
Anthozoa Corallimorpharia (3)	C1, C3c	[301], [305]
Scyphozoa (2)	A1, A3, B1, C1	[301], [305]
Hydrozoa (3)	A3, A4, A4a, B1	[301], [305]
Gorgonaceae (21)	B1, B1a, B1b, B8, B9, B19, C1, C3	[301], [305]
Gastropoda (1)	B1, C4	[305]
**Total species = 85**	**Total clades = 31**	**Total references = 17**

**Note:**

Numbers in parentheses beside the taxonomic group represent the number of species within that group reported to have symbiosis with *Symbiodinium* clades (See Table S2 for the complete list of species known to have different clades of *Symbiodinium* as symbionts).

**Table 4:** Number of Caribbean species of sponges (Spon), scleractinian corals (Cor), mollusks (Moll), amphipods (Amph), and echinoderms (Echi), per kilometer of coast per country within the five ecoregions.

Ecoregion/country	Spon	Cor	Moll	Amph	Echi	Total species	Coastline length (km)	Species/100 km
**WESTERN CARIBBEAN**	**243**	**73**	**938**	**142**	**268**	**1664**	**2089**	**80**
*Mexico (Yucatán)*	118	63	733	133	182	1229	911	120
*Belize*	193	51	580	24	134	982	386	248
*Honduras*		62	580		95	737	644	114
*Guatemala*		27			23		148	34
**SOUTH-WESTERN CARIBBEAN**	**222**	**81**	**1451**	**91**	**284**	**2129**	**3880**	**55**
*Colombia*	142	65	1168	63	180	1618	1880	83
*Panama*	146	62	587	21	155	971	1295	73
*Nicaragua*		41	129		65	235	493	48
*Costa Rica*	64	47	638	21	23	793	212	364
**SOUTHERN CARIBBEAN**	**225**	**87**	**944**	**208**	**151**	**1615**	**3444**	**47**
*Venezuela*	144	79	664	195	124	1206	2722	37
*ABC* *	113	68	239	20		440	360	117
*Trinidad & Tobago*		41			55	96	362	27
**GREATER ANTILLES**	**335**	**91**	**1943**	**164**	**248**	**2781**	**8477**	**33**
*Jamaica*	169	72	824		86	1151	1022	113
*Cayman Islands*	82	62	477			621	160	388
*Puerto Rico*	40	72	1078	25	121	1336	501	262
*Cuba*	255	72	1300	131	145	1903	3735	47
*Hispaniola*	71	72	572	16	117	848	3059	27
**EASTERN CARIBBEAN**	**126**	**71**	**1119**	**46**	**79**	**1441**	**1322**	**109**
*Lesser Antilles*								

*(ABC =  Aruba, Bonaire, Curacao).

The number of endemic species could be established with relatively high confidence for only 21 of the 78 higher taxa (27%) (Table S1). The total number of endemic species for those taxa is 1,563, which represents 25.6% of the species for these groups. However, this estimate of endemism cannot be extrapolated to the whole Caribbean biota, because the relative contribution of the different taxa varies strongly. For example, about 45% of the fish species are considered Caribbean endemics, whereas endemism in mollusks amounts to about 26% and in copepods to only 2%. Notable differences are also apparent between closely related groups, such as the proportions of endemics among the bivalves (17.9%) and Gastropoda (29.3%), as well as those among Amphipoda (1.3%), far lower than that among Copepoda (9.2%). Note that these estimates hold only for the Caribbean Sea as defined above and not for the so-called Greater Caribbean, which also encompasses the Gulf of Mexico, Florida, the Bahamas, and Bermuda. Since the Caribbean shares many species with these adjacent regions, each of which has its own endemics, estimates of endemism for the Greater Caribbean are likely to be higher.

**Figure 2:**
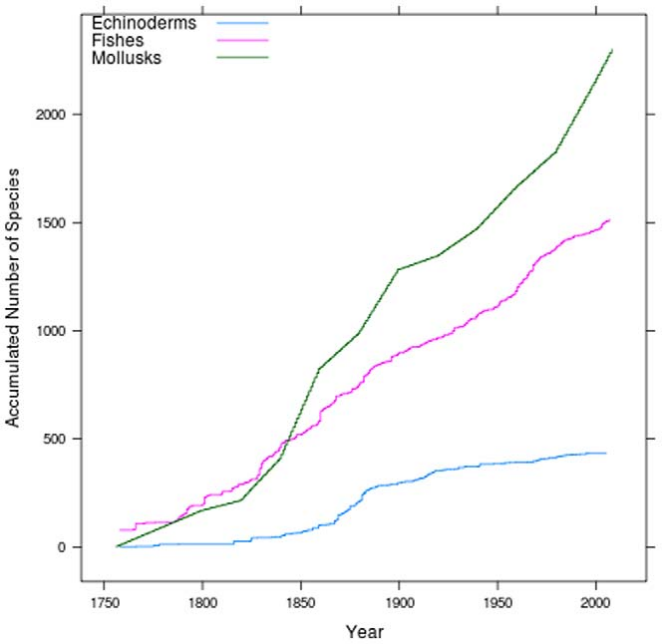
Species-description accumulation curves for Caribbean mollusks, echinoderms and fishes.

**Figure 3:**
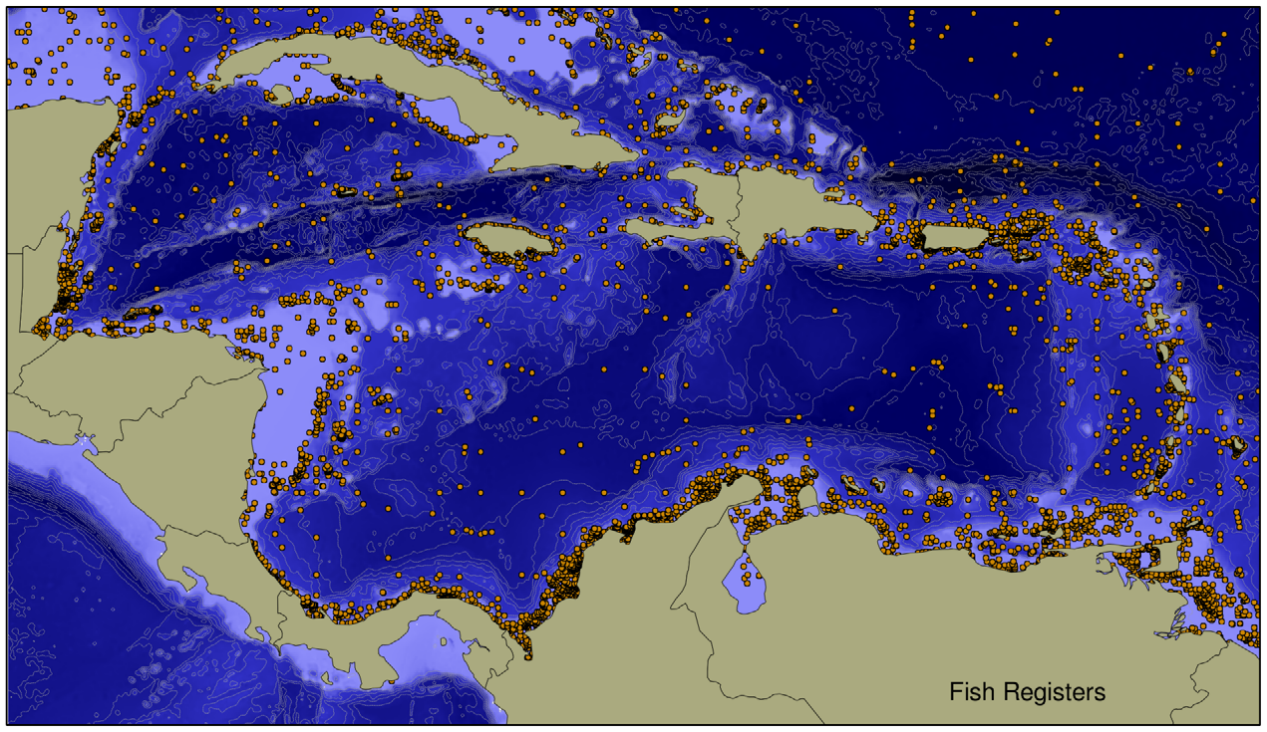
Geographic distribution of shallow water bony fishes and elasmobranchs. Geographic distribution of 161,000 records of shallow water bony fishes and elasmobranchs in the Greater Caribbean (Caribbean proper plus the Bahamas, the Gulf of Mexico, Florida, and Bermuda). Data were drawn from 20 museum databases, 9 Web databases, and 98 publications.

With the exception of mangroves, seagrasses, mammals, birds, and reptiles, we can expect that the number of species recorded in the Caribbean will increase in the future for the majority of taxa, particularly for those groups scored lower than 3 for “state of knowledge” in Table 2 and Table S1. However, even for relatively well known groups, such as mollusks, echinoderms, and fishes, the inventories have by no means been completed, and further discoveries (descriptions of new species or first Caribbean records of known species) ought to be expected. For relatively well known and not very species-rich groups, such as echinoderms, the accumulation curve of species discovery in the Caribbean shows that it is approaching an asymptote. In contrast, the accumulation curves of species-rich groups, including mollusks and fishes (Figure 2), suggest that a full inventory of these taxa is still far from being completed and that, despite the long history of collecting in a relatively small area, there are still many species to be discovered. As an example that supports this assertion, the map on Figure 3 shows the spatial distribution of 161,000 datapoints representing historical fish records in the Greater Caribbean, which represent 2,927 areas or localities of 10×10 km. That distribution indicates that within this region, large areas, even along the coastal zones, are seriously undersampled. Those areas include a large portion of Cuba, the large area of continental shelf off Nicaragua and Honduras, the ocean banks between Nicaragua and Jamaica and between Honduras and the Caymans, all of Hispaniola, the extreme northeastern Lesser Antilles, and some of the reefs offshore from Venezuela. In general, sampling effort has been best for shallow nearshore waters, where there is relatively good coverage of species records, especially along the southern Caribbean coasts (Belize, Costa Rica, Panama, Colombia, and Venezuela), in Puerto Rico, and much of the Lesser Antilles.

**Figure 4:**
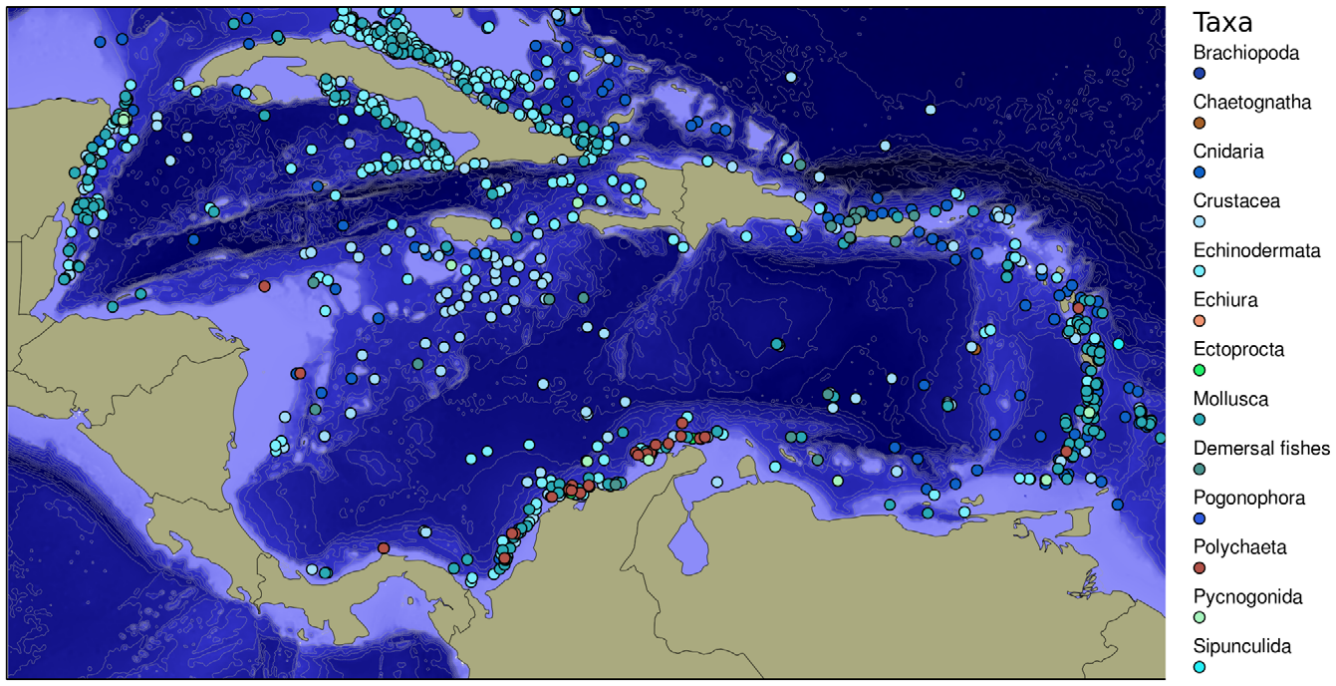
Distribution of deep-water species records (>200 m) in the Caribbean Sea.

The collecting effort in settings deeper than 200 m has been concentrated along the Mexican and Colombian continental slopes and abyssal plains, the north and south coasts of the eastern two-thirds of Cuba, the south coast of Jamaica, and the Lesser Antilles arc. Elsewhere in the Caribbean, records are much more sparse and scattered. Very few records exist for areas between Honduras and Panama, along the shelf north of Venezuela, and off western Cuba (Figure 4). The Caribbean basin deep-sea species database includes 1,530 species grouped in 12 phyla: Porifera, Cnidaria, Chaetognata, Mollusca, Sipunculida, Annelida, Bryozoa/Ectoprocta, Brachiopoda, Pycnogonida, Crustacea, Echinodermata, and Cephalochordata (demersal fish only). Further, the data derived from these distributions of sampling effort in the deep sea, even in relatively heavily sampled areas, are limited by the fact that different sampling methods were used in different areas and that the long history of collecting has occurred in bursts of activity in different places at different times.

For many species groups in the Caribbean, the only currently active taxonomists work in universities, museums, or research institutions outside the region. Current local expertise is completely lacking for several important taxa, particularly those with small body sizes and little economic significance, such as Mesozoa, Nemertea, Phoronida, Brachiopoda, Pogonophora, Kinorhyncha, and Chelicerata. The best-covered taxa with local expertise are Angiospermae, Aves, Reptilia, and Pisces, and moderate capacity exists for Porifera, Polychaeta, and some groups of Algae, Crustacea, Mollusca, and Cnidaria (Table 2). Moreover, only a small fraction of the local experts are employed as full-time systematists or taxonomists. For several groups, the coverage of available guides and identification keys for known species is good (marine mammals, fishes, turtles, birds, reef corals, reef sponges, ascidians, mollusks, amphipods, algae), although some are outdated. However, for many other groups, such guides are either inadequate or completely lacking (Table 2). One of the most recent regional taxonomic guides in the region is on shallow-water ascidians and includes descriptions and photographs of living animals [60].

### Geographic patterns of species richness

**Figure 5:**
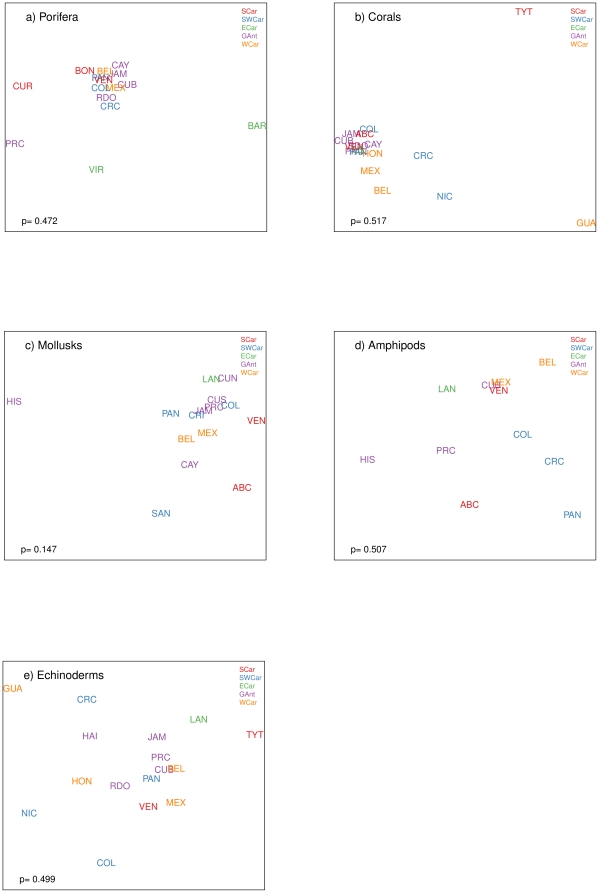
MDS for various taxa between the different Caribbean countries or subregions. SCar: Southern Caribbean (red), SWCar: Southwestern Caribbean (blue), ECar: Eastern Caribbean (green), GAnt: Greater Antilles (purple), WCar: Western Caribbean (orange).

The species of sponges (Table S3), scleractinian corals (Table S4), polychaetes (Table S5), mollusks (Table S6), amphipod crustaceans (Table S7) and echinoderms (Table S8) were compiled for the different countries or subregions within the Caribbean. Spatial patterns of species diversity usually exhibit relatively definitive gradients or shift progressively in space, unless ecological factors change abruptly. We expected to find species composition to be more similar between countries within one ecoregion in relation to countries within a different ecoregion or with areas located farther apart, however, this was not observed (Figure 5). The MDS ordination of the species by country within ecoregions is very different from one taxonomic group to the other, and no signicant differences were found in species composition between ecoregions for any of the taxonomic groups. MDS stress values for the figures were very low (0.005–0.129), indicating that the 2-dimensional plots are a good representation of the data [61].The species composition of sponges throughout the Caribbean is relatively homogenous with the exception of Curacao, Puerto Rico, Virgin Islands, and Barbados. Barbados and the Virgin Islands are both from the Eastern Caribbean region, and despite being different in composition from the rest, they are also different from each other (Figure 5a). The same tendency of species homogeneity throughout the Caribbean can be observed for corals, with the exception of Trinidad and Tobago and Guatemala (Figure 5b). For mollusks, species composition was similar within several countries from the Greater Antilles ecoregion (with the exception of Hispaniola island), for the Western Caribbean, for the Southwestern Caribbean (with the exception of San Andres Island), and for the Southern Caribbean. The Eastern Caribbean was grouped closely with all of the ecoregions except for the Southern Caribbean (Figure 5c). In the case of amphipods, Cuba, Mexico, and Venezuela are closely grouped together which is probably an artifact due to the fact that these three countries are the best sampled in the Caribbean with extensive species list for amphipods (these countries list more than 130 amphipod species, while the rest list between 16 to 63 species only). For this group, species composition is relatively similar within the Western Caribbean ecoregion and within the Southwestern Caribbean ecoregion (Figure 5d). In the case of echinoderms, species composition was relatively similar within the Greater Antilles ecoregion, and within the Western Caribbean ecoregion (Figure 5e). In terms of absolute species richness by ecoregion, for these groups, the most speciose ecoregion was the Greater Antilles with 2781 species, followed by the Southwestern Caribbean with 2129, the Western Caribbean with 1664, the Southern Caribbean with 1615, and finally the Eastern Caribbean with 1441 species (Table 4). The Greater Antilles is also the most speciose region for sponges, corals, and mollusks, while amphipods were more diverse in the Southern Caribbean, and echinoderms in the Southwestern Caribbean. A very large proportion of the species in this compilation is from coastal shallow waters, therefore, coastal length was considered within each of the ecoregions. When the species richness is viewed in terms of species per 100 kilometers of coast, the situation is different: the Eastern Caribbean has the highest number of species per coastal length (109 species/100 km of coast), followed by the Western Caribbean (80), the Southwestern Caribbean (55), the Southern Caribbean (47), and finally the larger area, the Greater Antilles (33 species/100 km of coast) (Table 4). When looking in detail at biodiversity richness within each of the ecoregions, the Porifera are clearly more species rich (165–255 species) in Cuba, Belize, and Jamaica than elsewhere in the Caribbean. This group is significantly less diverse (40–85 species) in Hispaniola, Puerto Rico, and the Lesser Antilles, as well as along the Nicaraguan and Costa Rican coasts. Intermediate richness values (113–146 species) occur in Yucatan, southern Central American, and northern South American coasts, including the Leeward Antilles (Aruba, Bonaire, and Curacao, or ABC Islands, and Venezuelan offshore islands). The Cayman Islands have only 82 reported species, however, the Cayman Islands are small coral islands, and as measured by the number of sponge species per kilometer of coastline the Cayman Islands and the ABC Islands rank as the most species-diverse areas in the Caribbean (Table 4). With regard to zooxanthellated hard corals, species-rich areas (containing more than 70% of all Caribbean species) occur throughout the region, but Hispaniola and Venezuela clearly stand out with 63 and 68 species, respectively. On the contrary, Guatemala, Nicaragua, Costa Rica, and Trinidad and Tobago are less diverse areas. Again, considering the number of species per kilometer of coastline, the Cayman Islands and ABC Islands are by far the most species-diverse areas. On the other hand, azooxanthellate corals, most of which occur in deeper waters and have not been thoroughly surveyed in many areas, are apparently more diverse in Cuba, Jamaica, and Trinidad and Tobago.

Among the Mollusca, the Gastropoda appear to be more diverse (more than 750 species) in Cuba, the Lesser Antilles, and Colombia. The Bivalvia exhibit a similar trend but seem by far more diverse in Puerto Rico (308 species) than in the Lesser Antilles (248 species). The Polyplacophora are conspicuously more diverse (23–28 species) along the Greater and Lesser Antilles and in Colombia than along the Central American coast and in other Caribbean islands (fewer than 18 species). Species numbers of Scaphopoda and Cephalopoda vary greatly between countries, but in both cases they appear to be more diverse in Puerto Rico (33 species) and Colombia (23 species). In general, mollusk species richness seems to be highest (more than 1,000 species) in Cuba, Colombia, the Lesser Antilles, and Puerto Rico. Intermediate richness occurs in Jamaica, Yucatan, Belize-Honduras, Costa Rica, Panama, Venezuela, and Hispaniola, and least species richness occurs in Nicaragua and around ABC, the Cayman Islands, and other oceanic small islands in the central Caribbean. Species richness among Echinodermata is high in Yucatan and Colombia (more than 180 species), intermediate (117–155 species) in Venezuela, Panama, Belize, Cuba, Hispaniola, Mexico (Yucatan coast) and Puerto Rico, and rather low (more than 95 species) in Trinidad and Tobago, the Lesser Antilles, and Jamaica and along the Central American coast.

The highest numbers for all-taxa species richness are found in Cuba, Colombia, the Lesser Antilles, and Puerto Rico, with intermediate richness in Venezuela, Yucatan, Jamaica, Belize, and Panama, and relatively low richness in the other countries. However, when the number of species of sponges, corals, mollusks, and echinoderms is combined and standardized by length of coastline (Table 4), the highest numbers of coastal species per kilometer of coastline occur in the Cayman Islands, followed by the ABC Islands, Costa Rica, the Lesser Antilles, and Puerto Rico.

**Figure 6:**
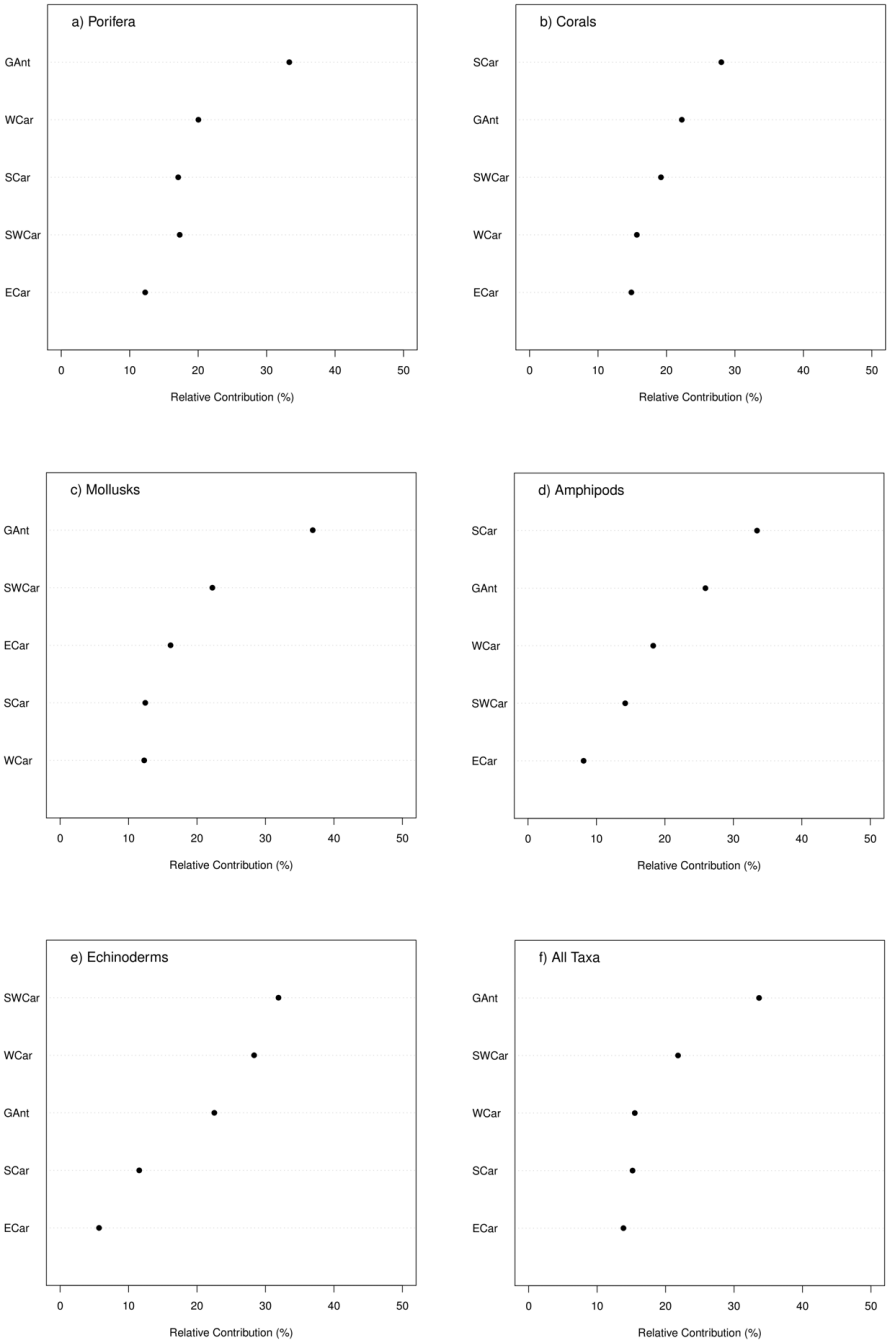
Contributions from Caribbean ecoregions to regional species diversity (gamma diversity) for five taxa. For each taxonomic group, the ecoregions are ordered by alpha diversity, from higher to lower. SCar: Southern Caribbean, SWCar: Southwestern Caribbean, ECar: Eastern Caribbean, GAnt: Greater Antilles, WCar: Western Caribbean.

**Figure 7:**
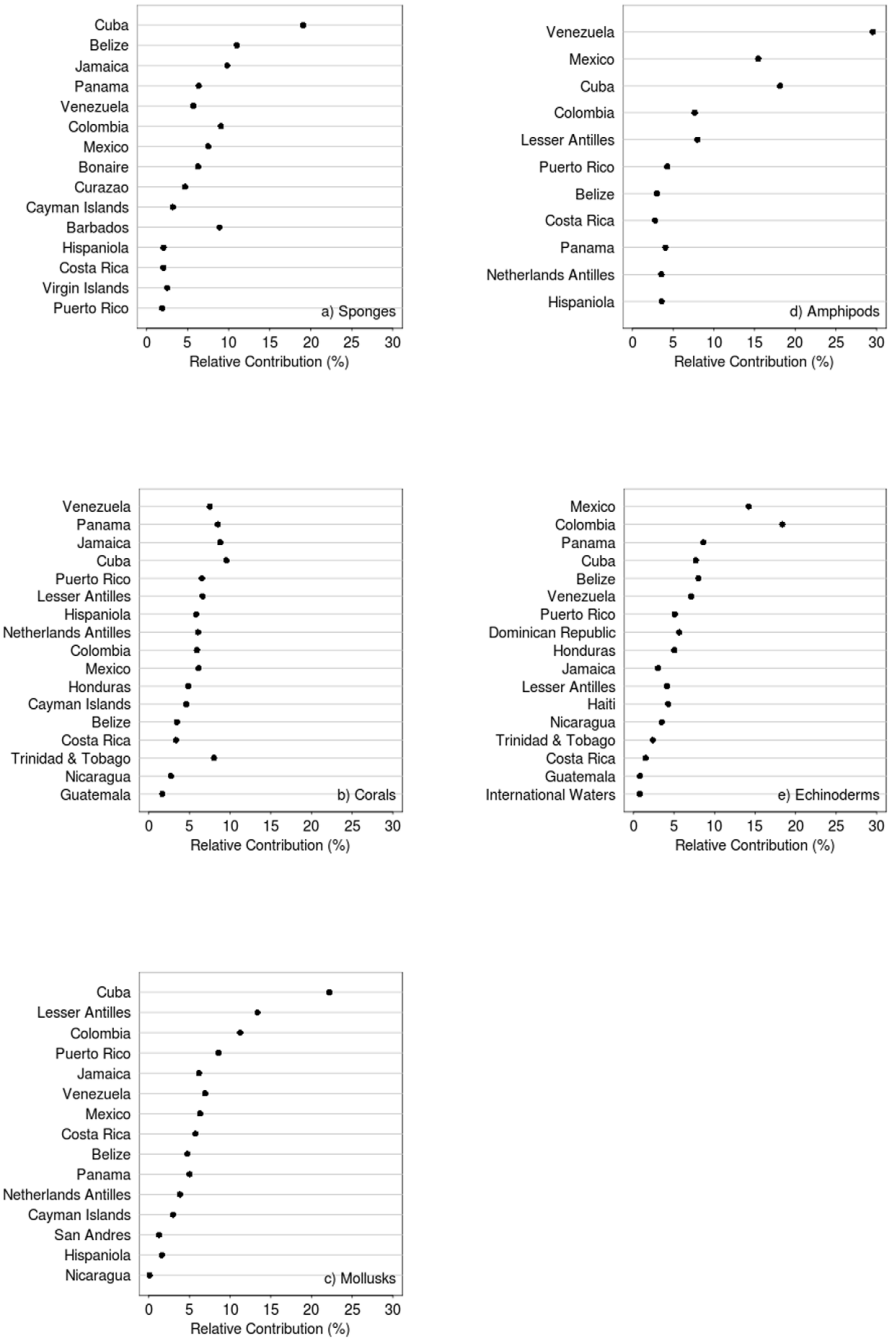
Contributions from individual Caribbean countries to regional species diversity (gamma diversity) for five taxa. For each taxonomic group, the countries are ordered by alpha diversity, from higher to lower.

While counts of species numbers may reasonably reflect the biological richness of a given area, they do not reflect its uniqueness. The latter is an equally significant measure of an area's importance in a wider context. A useful measure of an area's uniqueness is the number of endemic species it contains or of species that are likely to occur only in this area within the region but are more or less widely distributed in other regions outside the evaluated region. To measure uniqueness, the relative contribution of local (by country or subregion) diversity (alpha diversity) to the regional diversity (gamma diversity) was assessed. Figure 6 presents for five taxa: sponges (Figure 6a), hard corals (Figure 6b), mollusks (Figure 6c), amphipods (Figure 6d), echinoderms (Figure 6e), and for all taxa combined (Figure 6f) the relative contribution of species diversity from the five Caribbean ecoregions to the whole species diversity in the Caribbean region (gamma diversity). For each taxonomic group in the figure, the ecoregions are ordered by alpha diversity. For all of the five groups (Figure 6a–6e), the regions that had the higher alpha biodiversity were also those that contributed more to the regional (gamma) diversity, however, the contribution by ecoregion was different depending on the taxonomic group. The Greater Antilles is the ecoregion that contributes more to the region's diversity when all species from the five taxonomic groups are combined, a trend that was also observed for sponges and mollusks. For corals and for amphipods, the Southern Caribbean was the most contributing ecoregion, while for echinoderms, it was the Southwestern Caribbean. The ecoregion with the lowest contribution to the region's gamma diversity was the Eastern Caribbean. These ecoregional trends, however, may hide important contributions from smaller areas. When smaller areas within ecoregions were studied in detail, some countries also showed particular endemisms (Figure 7). In general, countries with a higher number of species also contribute more to the regional diversity (e.g., Cuba for sponges, Venezuela for amphipods, Mexico and Colombia for echinoderms). Nevertheless, there are some exceptions. In Barbados, for example, the number of sponge species (alpha diversity) is not very high, and as a country, it ranks in the bottom 30% of Caribbean countries for this group. However, its species seem to contribute significantly to the regional, gamma diversity, even more than Panama and Venezuela, which rank among the top 30% of countries with high diversity.

Cuba not only is the most diverse country for the Porifera but also has the most unique sponge composition by far (Figure 7a). Regarding zooxanthellate corals, despite Cuba not having the highest diversity, it is the most important contributor to the regional diversity, closely followed by Jamaica, Panama, Trinidad and Tobago, and Venezuela, the last of these being the most diverse in species number (Figure 7b). For Mollusca, the plot exhibits a nearly straight correlation between alpha diversity and its contribution to gamma diversity. Cuba (north and south), the Lesser Antilles, and Colombia, are not only the most diverse countries or subregions but also the major contributors to Caribbean mollusk diversity (Figure 7c). Venezuela, Cuba, and the Mexican Caribbean are clearly the major contributors to the regional species diversity of Amphipods, whereas the contribution of the remaining countries is rather low (Figure 7d). In Echinodermata, alpha diversity and its contribution to regional diversity are highly correlated; the Mexican and the Colombian Caribbean are the most diverse as well as being the major contributing subregions (Figure 7e).

**Figure 8:**
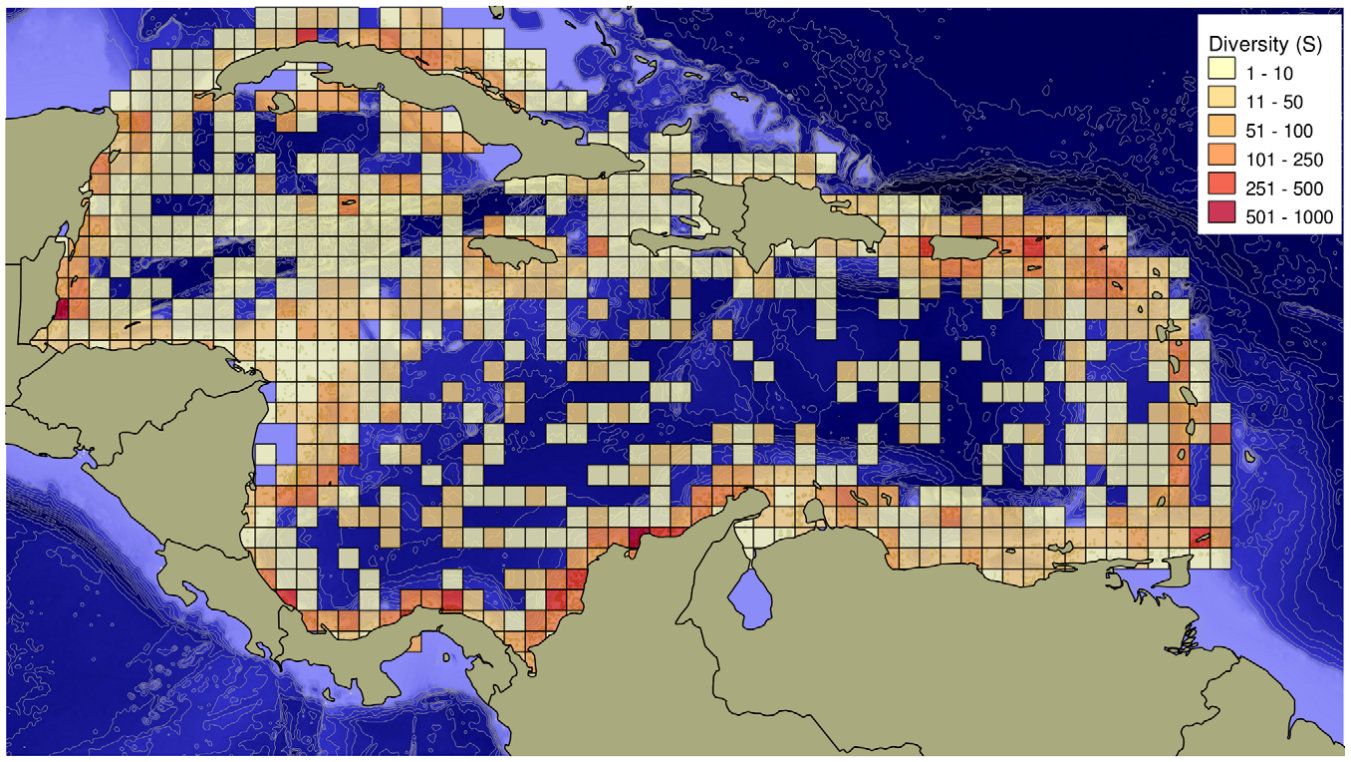
Spatial distribution of sites (small dots) and number of species recorded (squares) in the Caribbean. Based in data contained in the OBIS (Ocean Biogeographic Information System) database. Taxa included in the OBIS database were: bacteria, protozoa, microalgae, macroalgae, angiosperms, sponges, cnidarians, gnathostomulids, nematodes, kinorynches, sipunculans, mollusks, annelids, pogonophorans, arthropods, brachiopods, chaetognaths, echinoderms, tunicates, lancelets, fishes, reptiles, birds, mammals.

A major problem with these data on more than 12,000 species is that spatial locations are unknown for many, and thus species distributions cannot be mapped. To visualize marine diversity distribution patterns in the Caribbean, we relied on the OBIS database, which includes about 50% of the species reported here for the Caribbean (Figure 8). This map shows very clearly that biodiversity is concentrated around areas with a long history of research: Cuba, Colombia, Belize, Panama, Puerto Rico, and Tobago.

### Threats to biodiversity: Invasive species

Except for a couple of species—the green mussel (*Perna viridis*) and the red lionfish (*Pterois volitans*)—little is known or documented on the status of marine alien species. A total of 45 alien species belonging to 17 of the 78 taxa groups are known to date (Table 2). The most important introduced taxa in numbers of species are the Pisces (15 species), Crustacea (7 species), and Mollusca (6 species). The absence of records of introduced species in other groups is indicative of the poor level of taxonomic knowledge and does not necessarily signify a lack of introduced species. In addition, there is often difficulty in deciding whether newly reported marine species are introduced aliens, or native species that had not been formerly recorded. In sponges for instance, 10% of the species listed here have dubious taxonomic status because they were described originally from other biogeographic regions (Mediterranean, Northern Atlantic, Arctic, or Pacific). Only careful taxonomic comparisons, and in some cases genetic studies can help to discern whether these species are endemic, invasive, or simply distributed over wide ranges.

## Discussion

### Taxonomic inventories

Biodiversity assessments are fundamental not only for basic diversity science from the ecological, biogeographical, and evolutionary perspective, but also for ecosystem and ocean management as well as for the establishment of conservation policies. A recent regional example of a biodiversity assessments was carried out at the Saba Bank (Netherland Antilles) in fish [62], [63], macroalgae [64], sponges [65], hard corals [66], and octocorals [67]. Results of this research highlight the importance of habitat heterogeneity and the relative richness of the marine flora and fauna of the Saba Bank as targets for conservation [68].

Despite a long history of taxonomic research in the Caribbean, the marine biota of the region remains far from well known. The current record of approximately 12,000 marine species is clearly an underestimate for such a large and environmentally diverse tropical region. The same number of marine macroscopic species has been recently estimated for the Mediterranean Sea [69], [70], a temperate marine region of similar size to the Caribbean. The ability to develop a more accurate inventory of Caribbean species is hampered by the lack of comprehensive regional identification guides for most taxa, limiting the ability to make accurate species inventories and more thorough revisions of most taxa. In addition, the ability to make revisions is limited by the fact that many of the collected specimens are not deposited in local collections but remain scattered worldwide. In this sense, the Caribbean is no exception to the general problem of limited taxonomic expertise. The main goal of the Census of Marine Life program was to study the diversity distribution, and abundance of marine life. For this, the program used two approaches, the first was through exploration to new areas and ecosystems, and the second was through a review of the known. In both cases, the program achieved to coordinate and centralize all the information that was scattered all over the world, largely in unavailable formats and sources. Regardless of the approach used, taxonomic expertise was a need, and this has been one of the major limits to knowledge. In the last decade, molecular techniques have been refined and certainly provided a method to address many of the challenges of assessing diversity. In coral reefs around the world, including in the Caribbean, the Census CReefs project coupled the use of Autonomous Reef Monitoring Structures or ARMS with environmental genomics to assess the enourmous diversity in these systems as well as to monitor understudied coral reef invertebrate biodiversity, along with the effects of climate change and ocean acidification [71]. Monitoring and biodiversity assessments will allow us to better understand diversity patterns and will improve the effectiveness of management strategies for marine ecosystems. To do so, capacity building in taxonomy through molecular techniques and monitoring is essential. Inevitably, given the limited number of active taxonomists within the Caribbean region, while some taxa have received much attention (for example, fish, mollusks, corals, sponges, and some crustacean groups), many taxa have been completely neglected (most of the meiofaunal groups, the bacteria, and most of the protoctista). Sampling effort has also been strongly biased toward certain habitats in coastal and shallow waters, particularly coral reefs, with very little collecting of benthic organisms in waters deeper than 500 m, which cover more than 75% of the region. In addition, certain countries or subregions have been more exhaustively surveyed than others. For example, the apparently low species richness of most taxa in Hispaniola, Guatemala, Nicaragua, and several oceanic islands probably is an artifact of low sampling intensity. All of these factors contribute to taxonomic, regional, and habitat biases in the current state of knowledge of Caribbean biodiversity. Such biases are probably responsible in large part for the marked variations of species richness between countries or ecoregions and the apparent spatial inconsistencies in some of our similarity analyses. Most deep-sea studies in the Caribbean Basin have been carried out historically by scientific groups from other regions in the world. Deep-sea research capacity in the Caribbean is limited to laboratories that have the infrastructure and vessels to sample at the depths required. In the Caribbean, only Mexico, Colombia, and Venezuela have such capabilities. Outside the Caribbean, additional countries include France, Germany, Great Britain, and the United States. Thus, many historical records were obtained through large international research efforts. Examples of these records are the sponge surveys in Barbados. The Barbados data were obtained mostly from Soest and Stentoft [72] and are restricted to a collection from sponges dredged at deep waters. Another unprecedented collection effort in the Caribbean was the shallow-water and submersible collections (with the *Argus* in 1984 and RV *Seward Johnson* in 1997) of Hexactinellida and Lithistid (Demospongiae) in Cuba.

The number of scientists who have expertise in deep-sea Caribbean taxa does not exceed 15. As many of these experts work at major museums in the United States and Europe, the local and regional scientific capacity for such research is very low.

Sampling effort has clearly been best for shallow, nearshore waters, especially along the southern Caribbean coasts (Belize, Costa Rica, Panama, Colombia, and Venezuela), off Mexico, in Puerto Rico, and in some of the Lesser Antilles (Figures 2 and 8). Figure 8 shows that the areas with the highest known biodiversity in the region are in Belize and central Colombia. This pattern, however, may not reflect the true situation for regional Caribbean marine biodiversity for the following reasons: (1) the lack of collecting exhaustively in many areas or ecosystems (such as the deep sea and even coastal rocky shores), (2) the high variability between collecting methods, (3) the limited taxonomic expertise for many groups in the Caribbean, and (4) the very limited or inconsistent effort to make the data available through open-access digital databases. So, in the example of Belize and Colombia, the high species richness shown in Figure 8 is most likely a sampling artifact as the U.S. National Museum of Natural History (US-NMNH) for Belize, and Colombia's Institute of Marine and Coastal Research (INVEMAR), have carried out excellent surveys and made the data publicly available. Similar, sampling-intensity biases undoubtedly contributed to high relative diversity evident at other locations, including Puerto Rico and the Virgin Islands, Los Roques (Venezuela), the Cayman Islands, Panama and Costa Rica, the Colombian islands offshore from Nicaragua, and tiny Navassa Island, a U.S. territory between Jamaica and Hispaniola. Cuba provides a particularly instructive example, with enormous variation in apparent diversity around different parts of this large island. For example, Cuba has a high diversity of sponges (255 described species), but such high diversity is most probably a consequence of the significant deep-water exploration mentioned earlier (Figure 8).

A good example is the recently published compilation of Costa Rica's marine biodiversity, which includes exhaustive lists of all recorded species of many groups [73]. Detailed mapping of habitat types on national scales have also been undertaken in most of these taxonomically best-known areas: Colombia [74], [75], Guadalupe [76], Puerto Rico and the Virgin Islands [77], and Panama [78]. Conservation planning has also benefited greatly from both the relatively good knowledge of the species and the availability of habitat maps, for example, in Colombia [55], and Venezuela [56]. Many non-Caribbean researchers either participate in regional sampling through collaborative projects and international programs or conduct work of their own. Because researchers resident in a particular Caribbean country may not be aware of research being done locally by external institutions, quantifying diversity becomes difficult if available data are not published. Much of the work done by non-Caribbean researchers is encountered only in the primary literature and is disconnected from regional plans. With the highly welcome development of OBIS, however, this is rapidly changing as more institutions develop georeferenced digital databases of their collections and contribute those databases to this clearinghouse for global data.

An example of the power of databases to provide maps of biodiversity distribution and to visualize the areas where more effort is needed is illustrated with fishes (Figure 3). The continuing upward trend of the curve of new species descriptions (Figure 2) clearly demonstrates that we have a long way to go yet regarding new species descriptions, even in a relatively well known group such as fishes. Further discoveries will not only take place in the deep sea, which has been a less explored area but also in shallow water reefs. As an example of this, there are three recent papers describing new shallow water cryptic fish species (gobies and blennioids, the two most speciose groups of fishes) from Honduras, Saba, and Belize [62], [79], [80]. Some of those species appear to have very limited ranges, and there are substantial parts of the Caribbean that have had very little sampling (e.g. Cuba and the large shelf east of Nicaragua). Forensic barcoding has been important in revealing this under-appreciated diversity. Barcoding requires the use of freshly collected material and assessment of that diversity requires comparison of bodies and DNA that will be available mostly through new collected material.

### Geographic patterns of species richness

The Caribbean as a whole constitutes one distinctive subregion of the Tropical North Western Atlantic Province [41], [44], [45], [81]. However, the Caribbean is far from being homogeneous biogeographically. Its complex geological history and the present-day geographic diversity in hydrologic, morphologic, and habitat regimes, have led to the recognition of several distinct biogeographic sectors. Several criteria have been used to define these sectors, ranging from purely taxonomic comparisons of the present-day biota between subregions [41], [43], [82], [83], paleobiogeographic considerations [84], arrays of ecoregions according to habitat distribution patterns and biogeochemical factors [44], [45], to expert-derived systems without a rigorous core definition [29]. Taxonomic-based regionalizations have mostly focused on differences in composition of single groups at the class to family taxa levels. For example, the relatively detailed records that already exist of crinoid echinoderms, selected families of reef fishes, certain gastropod families, and porcellanid crabs from the Caribbean have allowed reasonable assessments of their spatial patterns as well as zoogeographic affinities [43], [82], [85]-[88]. Even the relatively widely adopted system proposed by Briggs [41] was based primarily on the degree of endemism among shore fishes. However, at present there is no biogeographic regionalization system for the Caribbean that is based on exhaustive comparative analyses of the distributions of all taxonomic groups, nor even of a single group at the phylum level.

Despite the absence of common geographic patterns in the studied groups, it can be noticed that, in general, species richness in the Caribbean tends to concentrate along the Antillean arc (Cuba to the southernmost Antilles) and the northern coast of South America (Venezuela, Colombia). General support for such a pattern comes from the comparatively greater contribution of the alpha diversity of these subregions to the regional diversity. However, that statement must be qualified by the recognition that apparently lower numbers of species on the Central American continental coast and oceanic islands and banks in the central Caribbean are probably at least partially due to reduced sampling effort in those areas and the overall geographic distribution of sampling effort (Figure 8). Either way, analysis of biogeographic patterns of the Caribbean marine biota taken as a whole, or even at the phylum to order level, is still difficult and limited, owing to insufficient and geographically biased sampling and knowledge.

Within the relatively small and densely packed Caribbean basin, spatial patterns are seemingly controlled by a number of interacting environmental factors, the effect of which appears to be variable depending on the different life histories of the taxonomic groups. Sandin et al. [87] found that diversity of reef-associated fishes around the Caribbean islands is highly dependent on island area and isolation, though nearshore productivity might play an important role as well. Endemism and distribution patterns of many gastropods along the southern Caribbean shelf are thought to be controlled by high productivity and low temperatures linked to upwelling areas [43], [84]. These results highlight complex, but fundamental, mechanisms that underlie spatial patterns of biodiversity within the Caribbean.

The ecoregion system of classification proposed by Spalding et al. [29], which we tested here, defined ecoregions as “areas of relatively homogeneous species composition and likely determined by the predominance of a small number of ecosystems and/or distinct oceanographic or topographic features”. While we have no doubt that the ecoregions proposed for the Caribbean have indeed distinct oceanographic and topographic features (e.g. upwelling in the Southern Caribbean, geologically recent topographic barrier in the Southwestern Caribbean, the Orinoco influx in the Eastern Caribbean, etc), it is unlikely that they can be defined by species composition. To our knowledge, this paper is the first attempt to review all known marine biodiversity in the Caribbean and to produce comprehensive species lists. Moreover, this collection is the first global effort to compile and organize all known information about marine biodiversity in the world's oceans. In this way, it is not surprising that species distribution was not consistent with the ecoregional approach in most of the cases. The patterns we observed may be explained differently, depending on the taxonomic group and can be due to a combination of variables as described above.

#### Sponges

The pattern of sponge diversity is best explained by two facts. The first is the extension of the collecting effort and the second is the taxonomic follow-up on the collected samples. In collection effort, several countries have been explored not only in the coastal zone but also in deep areas (e.g. Barbados by Soest and Stentoft [72]; Puerto Rico by Laubenfels [89]; Virgin Islands as described by Schmidt in the 1800s and dispersed in several publications), therefore, their taxonomic composition is different than in those countries in which only shallow water samples have been collected. On the other hand, in Curacao, almost no deep sea sponges have been reported but collecting effort in shallow waters and taxonomic descriptions has been significant [90]–[92]. In taxonomic follow-up, Cuba and Belize have benefited from outstanding taxonomic experience. To have a clear and correct picture of regional biodiversity, both facts are essential. For instance, in Panama, sponge collection up to 30 m in depth has been significant at Bocas del Toro, but taxonomic identification of these samples has not been completed. Once this identification is completed, the number of sponges at this locality will probably increase by at least 100 species, of which about 20 will be new descriptions. Recently, 13 new species of sponges were described from coral reefs of the Netherlands Antilles and the Colombian Caribbean [93].

#### Corals

Coral composition seems to be similar throughout the Caribbean regardless of the ecoregion with the exception of Trinidad and Tobago, and Guatemala. These two localities have fewer species than the rest of the Caribbean, as well as a more limited development and cover. In Guatemala, for example, the coral reef area is very small, and under the influence of sediments (Punta Manabique). In this area, live species cover is low, and species composition has been reported to be atypical for the Caribbean [94]–[96], mostly dominated by sediment-resistant species [97]. Trinidad and Tobago, on the other hand, are the most southerly of the eastern Caribbean islands, on the edge of the South American continental shelf, and under the influence of the Orinoco River. Trinidad has marginal coral communities with only sediment tolerant coral species (Siderastrea and Porites), while Tobago reefs, despite being more remote, are also threatened by nutrient and sediment runoff from land clearing and coastal development, sewage pollution and climate change [98].

#### Mollusks

Except for the coasts of Yucatan in Mexico, and Belize in the Western Caribbean, no consistent pattern was observed in the distribution of mollusks (gastropods and bivalves), suggesting that the geographic distribution of these groups in the Caribbean is mostly controlled by habitat type and distribution (coral reefs, seagrass beds, mangroves, rocky shores, estuaries, etc.). However, some lower level molluscan taxa (genus, family) do show some clear trends in their distribution patterns. Such is the case for the families Volutidae and Conidae, which have a series of allopatric species that are distributed along the coasts of Central America and the north of South America [99]–[101]. Another example can be observed in species of the genera Cypraeidae, Marginellidae, Olividae and Columbellidae which have many endemic species in the Southern Caribbean [43], [102], [103] that apparently originated through several vicariant events related to the evolution of the Caribbean Sea [84], [104]. In this way, the eventual biogeographic patterns due to vicariance events that can be observed in certain taxa, can be masked when trying to analyze spatial patterns for the whole group of mollusks.

#### Amphipods

The amphipod distribution pattern is clearly biased due to sampling effort and taxonomic expertise. This is a group that is poorly known in the Caribbean with the exception of Cuba, Venezuela, and Mexico. In these three localities, the number of species reported surpasses 130, therefore, the probability of sharing species between these three sites increases, and this is probably the reason why they are grouped together. To have a better idea of the real distribution pattern of amphipod species, more assessments are needed, especially in the Eastern and Western Caribbean, as well as in the rest of the Greater Antilles. With this new information, different groups would possibly form in the MDS that would reflect the true similarity in species composition between localities.

#### Echinoderms

The Caribbean seems to be relatively homogeneous in echinoderm species composition possibly due to the current patterns in this semi-enclosed basin. Ophiuroidea is the most diverse class which can be explained by the variety of cryptic habitats that this region provides for the development and speciation of this group (Table S1). There has been a marked bias in the sampling effort throughout the Caribbean, and consequently, the countries that have carried out more research are those that have the highest species diversity (Mexico, Belize, Panama, Colombia and Cuba). Efforts have also been dissimilar within the different classes. In Belize, for example, the ophiuoroid fauna is rich and well known [105]–[107], and the holothuroids are well known in the Eastern Caribbean and the Greater Antilles [108]–[112]. The Southwestern Caribbean ecoregion is the richest ecoregion, closely followed by the Western Caribbean, in which the Mesoamerican Reef could be a hot spot.

The countries or subregions contributing most to the regional diversity, while not necessarily the most diverse in all taxa, are also apparently the most rich in endemic or geographically restricted species (Figures 6 and 7). In this regard, Cuba and the southern Caribbean, especially the Venezuelan coast, stand out. Owing to its location on the northern edge of the region, the northern coast of Cuba has a rich marine biota that includes not only typical Caribbean species but also elements from the biogeographically somewhat different Gulf of Mexico and eastern North America (Carolinian Province of Briggs [41]). Moreover, in northern Cuba we see the persistence of many species of mollusks that, during the Miocene to Pliocene, had more widespread Caribbean distributions [113]. Another area regarded as a refuge, probably related to the occurrence of cold and nutrient-enriched upwelling waters, lies on the northern South American coasts of eastern Colombia and Venezuela [43], [114]. In addition, the extreme southeast corner of the Caribbean, which includes eastern Venezuela, has the greatest potential to receive immigrants moving northward along the east coast of South America, and is known to host significant numbers of such species. Such refuges, as well as island chains like the Antillean arc, represent areas rich in endemism and of active speciation, particularly for taxa with limited dispersal capability [113]. Smith et al. [115] analyzed GIS (geographic information systems) data on the distributions throughout the Greater Caribbean (the Caribbean plus the Gulf of Mexico, Florida, the Bahamas, and Bermuda) of 987 species of fishes and 144 species of invertebrates (mollusks and crustaceans). For fishes they found two peaks in species richness, one at southern Florida and northern Cuba, and the other along the northern coast of South America. For the invertebrates, richness was greatest in those same two areas plus the Lesser Antilles. They attributed the fish pattern to the abundance at those two hot spots of both local endemics and species that occur primarily in the northern and southern reaches of the Greater Caribbean.

### Threats to biodiversity and conservation of marine life

Rising population densities and associated coastal development, increasing fishing pressure, agricultural and industrial activities, increased river sediment loading, introduction of alien species, and climate change are among the major identified sources of anthropogenic pressure on Caribbean marine life (see [21], [47]).

Caribbean coral reefs are already greatly degraded, having declined in some cases from more than 50% live cover to less than 10% cover over the last two decades [48]. This degradation is due to a combination of impacts, including damage by hurricanes, diseases, bleaching, pollution, sediment runoff, overfishing, climate change, as well as more directly through boat anchoring, setting of fish traps, groundings of ships, dredging activities, collecting of corals, and dynamite fishing. The effects of climate change are particularly dramatic for coral reefs, particularly through ocean acidification and increased temperature [116]–[118]. These changes will lead to an increased frequency and severity of coral bleaching events as well as to problems in the calcification process of many organisms [119], [120]. Temperature increases have also been related to an increase in coral diseases in *Acropora*, as well as in *Diadema antillarum*. The disappearance of these two structural species leads to changes in the structure, composition, and dynamics of coral reefs. Diseases are also known to lower coral fecundity affecting the potential of natural recovery [121], [122]. Terrestrial runoff has been reported to cause eutrophication gradients in Barbados, increase bacterial biomass in the Grand Cayman Island, and sedimentation in Costa Rica. These changes have induced alterations in the community structure, reduced species diversity and live coral cover, and increased bioerosion (see review by Fabricius, [123]). Most of the examples about the effects of terrestrial runoff in the Caribbean are very localized, as the examples mentioned above, and in occasions fail to communicate their significance to environmental managers. In this sense, the region would greatly benefit from (1) a large scale ecological study along different water quality gradients and (2) addressing science within a framework that is scientifically rigorous, but that can be understood by a broader public. A successful implementation of this approach was carried out at the Great Barrier Reef in Australia [124].

High population pressure in coastal areas has also led to the conversion of many mangrove areas to other uses, including infrastructure, aquaculture, rice, and salt production. Mangrove loss has been occurring at about 1% per year in areal cover since 1980. In other words, about 413,000 ha of mangroves have been lost in the Caribbean since then [47]. In many areas of the Caribbean, seagrass beds are being removed to “improve” bathing beaches and to allow access to shipping, or to lay pipes and other submarine structures. They are also being buried by sediments from nearby dredging and filling activities and in many enclosed bays are severely affected by excessive organic loading and hydrocarbons [125]. Such loss is important because those ecosystems are the most symbolic and species-rich shallow-water ecosystems in the Caribbean [45], [47]. In fact, coral reefs, mangroves, and seagrass beds represent an integrated and interacting set of ecosystems [126], and it is therefore necessary to consider them as one large, interdependent marine ecosystem with shared biodiversity [47].

The problem of marine invasive species has been recognized only recently in the Caribbean. In 2006, the Venezuelan Institute of Aquatic Spaces (INEA), which is the focal point for the International Maritime Organization (IMO) organized the first regional workshop for the Caribbean regarding ballast water control with the goal of establishing a plan of action. In this workshop, the situation of Venezuela, Bahamas, Colombia, Cuba, Panama, Suriname and the Netherlands was presented from a political perspective, and it was summarized in the report of the GloBallast program in Venezuela and the Caribbean, prepared by the INEA (http://globallast.imo.org/). In 2007, another regional workshop was carried out at the Harte Research Institute for Gulf of Mexico Studies regarding Gulf of Mexico and Caribbean marine invasive species. This workshop had a more scientific focus and provided a list of key issues, priorities and future directions for research and management in the subject at the regional level [127]. A recent book edited by Rilov and Crooks [128] provides a detailed compilation and revision of several issues related to biological invasions in marine ecosystems in terms of conservation issues, vehicles, ecological understanding, and establishment of invaders, as well as discusses management and geographic perspectives. Many areas have substantial information about invasive species and processes (e.g. South Africa, New Zealand, Australia, Europe, China, Korea, and Brazil), but there is very limited information when it comes to the Caribbean. The Caribbean Sea has many potential vectors for the introduction of alien species. These include the Panama Canal, a major global crossroads for ship traffic, and many active ports that provide links for movement of species in ballast water or ship hulls. On the other hand, the most publicized marine invasive species that arrived to the Caribbean, the lionfish, was introduced through other mechanisms (aquarium trade activities). Our account records only 44 introduced marine or estuarine species in the region. However, not all introduced species are known or have been documented as invasive. Indeed, the compilation by the International Union for Conservation of Nature (IUCN) Global Invasive Species Specialist Group database (ISSG, http://www.issg.org/database) only lists for the Caribbean Sea a total of 12 alien or invasive species (4 fishes, 3 bryozoans, 1 mussel, 1 crab, 1 coral, 1 jellyfish, and 1 dinoflagellate). Venezuela, as the major exporter of unrefined hydrocarbon products in the Caribbean, imports about 96 million t of ballast water every year. Although only five marine invasive alien species have been recently reported from this country [129], four of those are not yet included in the ISSG database. Here again regional deficiencies in taxonomic inventories are evident. Additionally, the actual identification of species as native or introduced and the difficulty in determining whether newly reported marine species are introduced or cryptogenic natives represents a major constraint.

Marine ecosystems in the Caribbean region are interlinked through the movement of pollutants, nutrients, diseases, and other stressors, which will undoubtedly cause further degradation. There is considerable change occurring within the region, and solutions require analyzing pros and cons of networks of marine protected areas (MPAs), studying connectivity issues, the cooperation of neighboring countries, as well as a better understanding of stressors and measures that can be taken to ameliorate them [130]. Currently there are at least 600 MPAs in the Caribbean region. These areas include, among the IUCN categories of protected areas, 22 strict nature reserves, 103 national parks, and 350 managed nature reserves (http://cep.unep.org/caribbeanmpa). Most of the region's MPAs are coastal or nearshore and are intended to provide some coastal zone management while allowing varying levels and types of extractive activity within them [131]. These MPAs incorporate samples of most major marine and coastal ecosystem types, of which three of the most important—coral reefs, mangroves, and seagrass beds—are particularly well represented. However, coral reefs in many MPAs have been and remain degraded by human impacts, including overfishing, sedimentation from land-based development, land-based nutrient pollution, and anchoring [132].

### Role of the Census of Marine Life in the Caribbean

Although all of the Census projects involved in this region produced scientific advances, the contributions of the historic (HMAP), nearshore (NaGISA), and database (OBIS) projects can be especially highlighted. The HMAP project in the Caribbean was focused on the early human impact on mollusk populations and aimed to understand ancient human–mollusk interactions at a global scale. This initiative produced a special volume of the British Archaeological Reports (Antczak and Cipriani [133]) comprising 19 papers focused on two major themes: environmental and bioecological aspects of human–mollusk relationships, and sociocultural aspects of this relationship. The 19 papers are global in scope and include data from several mollusk species from around the world. In the Caribbean, these historical and archaeological studies were focused on the early exploitation and symbolic use of the queen conch (*Strombus gigas*) [133], [134].

Another major contribution of the Census program in the Caribbean has been the NaGISA project, aimed to study hard-bottom algal and soft-bottom seagrass communities worldwide by using a series of well-distributed standard transects from the high intertidal zone to a depth of 20 m. In the Caribbean Sea, rocky shores had been commonly neglected, as coral reefs have been the main research focus. The few studies in rocky shore biotas have focused on specific groups, such as algae [135], sponges [136], mollusks [137], or crustaceans [138]. Few studies have addressed patterns of spatial or temporal distribution of rocky-shore organisms at the community level [139]–[144].

Tidal ranges in the Caribbean are small (20–75 cm) and, consequently, the typical zonations reported elsewhere do not occur in this region. In addition, changes of assemblages across rocky shores occur over short horizontal distances (2–6 m, depending on the slope). For different parts of the Caribbean, three distinct vertical zones have been described by different authors (Good [141] for the British Virgin Islands, Nuñez et al. [142] for Colombia, and Miloslavich et al. [145] for Venezuela), although species composition varies geographically within those zones, which have geographically consistent features. From the few studies that exist for the Caribbean region, it can be generalized that rocky shores in this region are dominated by foliose algae (but see [146] for exceptions reported outside the Caribbean in Bermuda). This distinct pattern contrasts with previous studies in tropical shores, which report a dominance of herbivore-resistant algal forms, such as turf-forming algae (e.g., Wallenstein and Neto [147] and encrusting algae [148]–[152]). It has been proposed that this distinct pattern in the Caribbean (rocky shores dominated by foliose algae) might be due to a negligible effect of herbivores on these assemblages [140], a situation very different from that thought to exist on the rocky shores of other tropical regions [141].

About 300–320 different species of benthic macroorganisms (>2 cm) are commonly found in rocky shores across the Caribbean, of which 50–60% are macroalgae. Most of these belong to the genera Acantophora, Bryopsis, Caulerpa, Dyctiota, Laurencia, Padina, Polysiphonia, and Sargassum. Despite the minimal attention that these systems have received in the Caribbean, the importance of understanding the structure and dynamics of rocky systems is growing as a consequence of massive changes that coral reefs have suffered over the past several decades [48], including an 80% drop in live coral cover in 25 years [49]. Such decreases in live coral cover have increased the availability of hard substrata, which consequently may result in a phase shift from coral-dominated communities, to hard-bottom systems dominated by algae (e.g., [153]–[155]). In addition, understanding these rocky systems in the Caribbean is important because the traditional models proposed to explain the underlying mechanisms that determine and structure these communities (e.g., Lubchenco [156]; Menge and Sutherland [157]; others reviewed in Menge and Branch [158]) might not work in this region because of its uniqueness (i.e., high degree of endemism, biodiversity hot spot, distinctive geological history, and oceanographic conditions).

The Census project NaGISA represents the first attempt to study these rocky systems on a large-scale basis, especially at this time when these systems are likely to increase in cover, owing to the sharp decrease of coral reefs. It is comparable in scope to other regional initiatives centered on coral reefs and the potential changes that they might undergo, for example, the Atlantic and Gulf Rapid Reef Assessment (AGRRA, http://www.agrra.org) and the Caribbean Coastal Marine Productivity (CARICOMP) programs.

The third significant contribution of the Census in the Caribbean has been the input of regional data in the Ocean Biogeographic Information System (OBIS). One of the major problems identified during the Caribbean Marine Biodiversity Workshop in 2004 was that only some data in the Caribbean existed in electronic format and that much of the data required taxonomic revision. The idea of incorporating data into an open-access biogeographic information system, such as OBIS, was well received by the scientific community because it could become a powerful tool to better protect and manage biodiversity in a region heavily dependent on tourism and fisheries. Currently, OBIS has 184,796 records for 6,040 species from the Caribbean. Among the records of Caribbean species, 27,000 records for nearly half those species were contributed to OBIS during the last three years by INVEMAR in Colombia, and the Universidad Simon Bolivar in Venezuela. Despite these significant contributions, however, there are still about 6,000 known Caribbean species for which data are not yet included in the OBIS database; hence, much work remains to be done. A critical example is data on the deep sea. Data in OBIS for species in the deep Caribbean basins represents only about 10% of the records included in this synthesis.

A tremendous amount of work is still needed in the Caribbean to get a clearer picture of species richness and marine biodiversity patterns. The Census has made a first effort to compile the available information and to make this information more accessible to the scientific community. In this way, it has also been able to indicate where there are the gaps in knowledge in this region, including knowledge regarding taxonomic groups, inventories, geographical areas, and different habitats. The legacy of the Census in the Caribbean, as well as the international cooperation it has established, will continue after the first phase of the program ends in 2010, as we expect many scientific accomplishments and discoveries in the next years. As data are produced from the different projects, OBIS will also continue to grow, facilitating more research and providing a conservation tool that will allow the development of new approaches to regional management of these coastal environments for future generations. As Fredrick Grassle, founder of the Census of Marine Life program and chair of its Scientific Steering Committee for many years, said in his foreword to the book *Caribbean Marine Biodiversity: The Known and the Unknown*, “The Caribbean Census of Marine Life plans to increase understanding of the species and populations that historically live in these seas. This will expand the expectations for a richer and more diverse marine environment—a return to the paradise that once was” [37].

## Supporting Information

Table S1 Diversity, state of knowledge, and expertise of all taxonomic groups within the Caribbean region. Sources of the reports: databases, scientific literature, books, field guides, technical reports. State of knowledge classified as: 5 =  very well known (>80% described, identification guides <20 years old, and current taxonomic expertise); 4 =  well known (>70% described, identification guides <50 years old, some taxonomic expertise); 3 =  poorly known (<50% species described, identification guides old or incomplete, no present expertise within region); 2 =  very poorly known (only few species recorded, no identification guides, no expertise); 1 =  unknown (no species recorded, no identification guides, no expertise). Taxonomic experts were defined as people with expertise in the description and identification of particular groups of marine species (i.e., taxa).
(0.03 MB XLS)

Table S2 Summary of free-living and symbiont clades of *Symbiodinium* spp. sampled in the Caribbean.
(0.16 MB DOC)

Table S3 List of sponge (Porifera) species of the Caribbean and countries by ecoregion where the species have been reported. Data compiled by Cristina Díaz.
(0.12 MB XLS)

Table S4 List of zooxanthelate coral (Scleractinia) species of the Caribbean and countries by ecoregion where the species have been reported. Data compiled by Ernesto Weil, Jorge Cortés, and Carolina Bastidas.
(0.03 MB XLS)

Table S5 List of polychaete (Polychaeta) species of the Caribbean and countries by ecoregion where the species have been reported. Data compiled by Judith Gobin.
(0.11 MB XLS)

Table S6 List of mollusk (Mollusca) species of the Caribbean and countries by ecoregion where the species have been reported. Data compiled by Juan Manuel Díaz and Patricia Miloslavich.
(0.57 MB XLS)

Table S7 List of amphipod (Amphipoda) species of the Caribbean and countries by ecoregion where the species have been reported.
(0.08 MB XLS)

Table S8 List of echinoderm (Echinodermata) species of the Caribbean and countries by ecoregion where the species have been reported. Data compiled by Juan José Alvarado.
(0.10 MB XLS)
