# Supplementary material for: Marine Biodiversity in the Caribbean: Regional Estimates and Distribution Patterns
Source: PLoS One. 2010 Aug 2;5(8):e11916. doi: 10.1371/journal.pone.0011916 (PMC2914069; doi:10.1371/journal.pone.0011916)
Supplement: Table S2 — Summary of free-living and symbiont clades of Symbiodinium spp. sampled in the Caribbean. (0.16 MB DOC) [file pone.0011916.s002.doc]

Table S2. Summary of free-living and symbiont clades of *Symbiodinium* spp. sampled in the Caribbean.

| **Free-living** |  |  |
| --- | --- | --- |
| **Substrate** | **Clade designation of symbiont** | **Reference** |
| Sediments around corals | C | [1] |
| *Halimeda* spp  (including *H. opuntia*) | A4, B184, C | [1] |
| *Lobophora variegata* | A4, B184, C | [1] |
| *Lobophora variegata*  *Amohiroa* sp. | B184, C | [1] |
| *Amphiroa* sp. | A4, B184, C | [1] |
| Sediments around corals | A4, B184, C | [1] |
| *Dictyota* | B184 | [1] |
|  |  |  |
| **Symbionts** |  |  |
| **Species** | **Clade designation of symbiont** | **Reference** |
| **Anthozoa Scleractinia** |  |  |
| *Acropora cervicornis* | A3, C1, C2, C12 | [2,3,4,5] |
| *A. palmata* | A3 | [2,3,4,5,6] |
| *Agaricia agaricites* f. *agaricites* | C3a | [2,3,4,5] |
| *Agaricia agaricites* f. *danai* | C3a | [5] |
| *Agaricia danae* | C | [2,3] |
| *Agaricia lamarcki* | C | [2,3,4] |
| *Agaricia tenuifolia* | C3a | [2,3,4,5] |
| *Agaricia humilis* | C3a, C1, D1a | [4,5] |
| *Agaricia fragilis* | C3a | [5,7] |
| *Astrangia danae* | B | [6] |
| *Cladocora arbuscula* | B1 | [5] |
| *Colpophyllia natans* | B6, B9, C1 | [3,5] |
| *Diploria clivosa* | B1, C | [2,3,5] |
| *Diploria labyrinthiformis* | B1, C | [2,3,4,5] |
| *Dipolria strigosa* | B1, C1 | [2,3,4,5] |
| *Dendrogyra cylindricus* | B1 | [4,5] |
| *Dichocoenia stokesi* | B1 | [4,5] |
| *Eusmilia fastigiata* | B1, C1 | [2,3,4,5] |
| *Favia fragum* | B1, C1 | [2,3,4,5] |
| *Isophylastrea rigida* | C3 | [2,3,5] |
| *Isophylastrea sinuosa* | C3e | [5] |
| *Leptoseris cucullata* | C3 | [2,3,4,5] |
| *Madracis miranilis* | B | [2,3,6,8] |
| *Madracis decactis* | B7 | [2,3,5,8] |
| *Madracis formosa* | B1 | [5,8] |
| *Madracis pharensis* | B | [8] |
| *Madracis senaria* | B | [8] |
| *Manicina areolata* | B1, C1 | [4,5] |
| *Meandrina meandrites* | A, B1 | [2,3,4,5,9] |
| *Montastraea annularis* | A, B1, C3, C12, D1a | [4,5,8,10,11,12,13,14] |
| *Montastrea cavernosa* | C3, C7 | [2,5,15] |
| *Montastrea faveolata* | A, B1, C7, C12, D1a | [5,10,11,13,14] |
| *Montastrea franksi* | A, B, C, D | [7,10,13] |
| *Mycetophyllia ferox* | C | [2,3] |
| *Mycetophyllia danaana* | C3c | [5] |
| *Mycetophyllia lamarckiana* | C3c | [4,5] |
| *Porites astreoides* | A4a, A3, B1, C | [2,3,5] |
| *Porites colonensis* | A, C1a | [2,3,4,5] |
| *Porites divaricata* | C9 | [2,3,5] |
| *Porites furcata* | A4, B1, C4 | [2,4,5] |
| *Porites porites* | C10 | [2,3,5] |
| *Porites divaricata* | C1, C9 | [4,5] |
| *Scolymia cubensis* | C11 | [5] |
| *Siderastrea siderea* | C1 | [2,3,5] |
| *Siderastreradians* | B5a | [4,5] |
| *Stephanocoenia michelinii* | A3, C | [2,3,4,8] |
| *Stephanocoenia intercepta* | A3 | [5] |
| **Anthozoa Zoanthidea** |  |  |
| *Palythoa caribaeorum* | C1, D1 | [4,5,16] |
| *Palythoa grandis* | C3 | [4,5] |
| *Zoanthus sociatus* | A4, A3, B1, C1 | [4,5] |
| **Anthozoa Corallimorpharia** |  |  |
| *Discosoma carlgreni* | C1 | [5] |
| *Discosoma sanctithomae* | C1 | [5] |
| *Ricordea florida* | C3c | [4,5] |
| **Anthozoa Actinaria** |  |  |
| *Aiptasia tagetes* | B1 | [4] |
| *Actinopus elegans* | B1 | [5] |
| *Basrtholomea annulata* | C1 | [5] |
| *Condylactis gigantea* | A3, A4a, C1 | [4,5] |
| *Lebrunia danae* | C1 | [4,5] |
| *Rhodactis (Heteractis) lucida* | C1 | [5] |
| *Stichodactyla helianthus* | A4a | [5] |
| **Scyphozoa** |  |  |
| Cassiopeia xamachana | A1, A3, B1, C1 | [4,5] |
| *Linuche unguiculata* | C1 | [5] |
| **Hydrozoa** |  |  |
| *Millepora alcicornis* | A4a, A3, B1 | [4,5] |
| *Millepora complanata* | B1 | [4,5] |
| *Myrionema ambionense* | A4 | [5] |
| **Gorgonacea** |  |  |
| *Briareun asbestinum* | B19 | [4] |
| *Erythropodium caribaeorum* | C3 | [5] |
| *Eunicea mammosa* | B1, B9 | [5] |
| *Eunicea tourneforti* | B1, C1 | [5] |
| *Eunicea laciniata* | B1 | [5] |
| *Eunicea.ciavigera* | B1, C1 | [5] |
| *Gorgonia flabellum* | B1 | [4,5] |
| *Gorgonia mariae* | B1 | [5] |
| *Gorgonia ventalina* | B1 | [17] |
| *Muricea muricata* | B1 | [5] |
| *Plexaura homomalla* | B1, C1 | [4,5] |
| *Plexaura flexuosa* | B1b, B8 | [5] |
| *Plexaura nutans* | B1a | [5] |
| *Plexaura homamalla* | B1a | [5] |
| *Pseudoplexaura wagenaari* | B1 | [5] |
| *Pseudoplexaura flagellosa* | B1 | [5] |
| *Pseudopterogorgia rigida* | B1 | [5] |
| *Pseudopterogorgia kallos* | B1 | [5] |
| *Pseudopterogorgia americana* | B1 | [5] |
| *Pseudopterogorgia elisabethae* | B1 | [18] |
| *Pterogorgia anceps* | B1 | [5] |
| **Gastropoda** |  |  |
| *Strombus gigas* | B1, C4 | [5] |

**References:**

1. Porto I, Granados C, Restrepo J, Sánchez J (2008) Macroalgal-Associated dinoflagellates belonging to the Genus *Symbiodinium* in Caribbean Reefs. PlosOne 3: 1-5.

2. Baker A (2003) Flexibility and specificity in coral—algal symbiosis: diversity, ecology, and biogeography of *Symbiodinium*. Annu Rev Ecol Evol Syst 34: 661-689.

3. Baker A, Rowan R (1997) Diversity of symbiotic dinoflagellates (zooxanthellae) in scleractinian corals of the Caribbean and eastern Pacific. Proc 8th Int Coral Reef Symp 2: 1301-1306.

4. Banaszak A, Santos M, LaJeunesse T, Lesser M (2006) The distribution of mycosporine-like amino acids (MAAs) and the phylogenetic identity of symbiotic dinoflagellates in cnidarian hosts from the Mexican Caribbean. J Exp Mar Biol Ecol 337: 131-146.

5. LaJeunesse T (2002) Diversity and community structure of symbiotic dinoflagellates from Caribbean coral reefs. Mar Biol 141: 387-400.

6. Rowan R, Powers D (1991) A molecular genetic classification of zooxanthellae and the evolution of animal-algal symbiosis. Science 251: 1348-1351.

7. Wilcox T (1998) Large subunit ribosomal RNA systematics of symbiotic dinoflagellates: morphology does not recapitulate phylogeny. Mol Phylogenet Evol 10: 436-348.

8. Diekmann O, Bak R, Stam W, Olsen J (2001) Molecular genetic evidence for reticulate speciation in the coral genus *Madracis* from a Caribbean fringing reef slope. Mar Biol 139: 221-223.

9. McNally K, Govind N, Thome P, Trench R (1994) Smallsubunit ribosomal DNA sequence analyses and a reconstruction of the inferred phylogeny among symbiotic dinoflagellates (Pyrrophyta). J Phycol 30: 316-329.

10. Rowan R, Knowlton N (1995) Intraspecific diversity and ecological zonation in coral algal symbiosis. Proc Natl Acad Sci USA 92: 2850-2853.

11. Rowan R, Knowlton N, Baker A, Jara J (1997) Landscape ecology of algal symbionts creates variation in episodes of coral bleaching. Nature 388: 265-269.

12. Diekmann O, Bak R, Tonk W, Stam J, Olsen J (2002) No habitat correlation of zooxanthellae in the coral genus *Madracis* on a Curaçao reef. Mar Ecol Prog Ser 227: 221-232.

13. Toller W, Rowan R, Knowlton N (2001) Zooxanthellae of the *Montastraea annularis* species complex: patterns of distribution of four taxa of *Symbiodinium* on different reefs and across depths. Biol Bull 201: 348-359.

14. Thornhill D, Xiang Y, Fitt W, Santos S (2009) Reef endemism, host specificity and temporal stability in populations of symbiotic dinoflagellates from two Ecologically dominant Caribbean corals. PlosOne 4: 1-12.

15. Billinghurst Z, Douglas A, Trapido-Rosenthal H (1997) On the genetic diversity of the symbiosis between the coral *Montastraea cavernosa* and zooxanthellae in Bermuda. Proc 8th Int Coral Reef Symp 2: 1291-1294.

16. Kemp D, Cook C, LaJeunesse T, Brooks W (2006) A comparison of the thermal bleaching responses of the zoanthid *Palythoa caribaeorum* from three geographically different regions in south Florida. J Exp Mar Biol Ecol 335: 266-276.

17. Kirk NL, Ward JR, Coffroth MA (2005) Stable *Symbiodinium* composition in the sea fan *Gorgonia ventalina* during temperature and disease stress. Biol Bull 209: 227-234.

18. Santos S, Gutierrez-Rodriguez C, Lasker HR, Coffroth M (2003) *Symbiodinium* sp. associations in the gorgonian *Pseudopterogorgia elisabethae* in the Bahamas: high levels of genetic variability and population structure in symbiotic dinoflagellates. Mar Biol 143: 111-120.
